# Clustered architecture of ipsilateral and interhemispheric connections in macaque ventrolateral prefrontal cortex

**DOI:** 10.3389/fncir.2025.1635105

**Published:** 2025-08-26

**Authors:** Danling Hu, Hangqi Li, Toru Takahata, Hisashi Tanigawa

**Affiliations:** ^1^Interdisciplinary Institute of Neuroscience and Technology, School of Medicine, Zhejiang University, Hangzhou, China; ^2^Key Laboratory for Biomedical Engineering of Ministry of Education, College of Biomedical Engineering and Instrument Science, Zhejiang University, Hangzhou, China; ^3^Women’s Hospital, School of Medicine, Zhejiang University, Hangzhou, China; ^4^MOE Frontier Science Center for Brain Science and Brain-Machine Integration, School of Brain Science and Brain Medicine, Zhejiang University, Hangzhou, China; ^5^National Key Laboratory of Brain and Computer Intelligence, Zhejiang University, Hangzhou, China

**Keywords:** ventrolateral prefrontal cortex, area 45A, retrograde tracing, clustered organization, transcallosal connections, macaque monkey

## Abstract

The fine-scale organization of intrinsic and extrinsic connections in the primate ventrolateral prefrontal cortex (VLPFC), a region essential for higher cognitive functions, remains poorly understood. This contrasts with, for example, the well-documented stripe-like intrinsic circuits of the dorsolateral prefrontal cortex (DLPFC). To elucidate the circuit architecture supporting VLPFC function, we investigated the spatial organization of connections targeting the caudal VLPFC (primarily area 45A) in macaque monkeys using multiple retrograde tracers. Analyzing the distribution of labeled neurons in flattened tangential sections revealed that laterally projecting connections within the same hemisphere formed distinct clusters, not only in the VLPFC but also in the DLPFC. These clusters often spanned multiple cortical layers, suggesting a columnar-like organization. The width (minor axis) of these clusters was approximately 1.2 mm. Similarly, contralateral callosal projection neurons were also arranged in clusters. Additionally, inputs originating from the superior temporal sulcus were found to arise from discrete clusters of neurons. Our findings demonstrate that both long-range ipsilateral and interhemispheric connections of the caudal VLPFC share a common, fine-scale clustered architecture. This study provides an anatomical framework for understanding the structural basis of information processing and interhemispheric coordination within this critical association cortex, suggesting that this architecture is fundamental to VLPFC’s role in complex cognitive functions.

## 1 Introduction

The ventrolateral prefrontal cortex (VLPFC), a region within the prefrontal cortex (PFC), plays an essential role in diverse higher cognitive functions in primates, subserving processes such as the active maintenance and selection of information in working memory, retrieval and comparison of memories, and the control of behavior based on abstract rules and context (Miller and Cohen, 2001; Owen et al., 2005; Petrides, 2005; Badre and D’Esposito, 2009). Anatomically, this region is not a single homogeneous functional unit but is known to comprise multiple subdivisions [e.g., Areas 45A/B, 12 (or 47/12), 46v] characterized by distinct cytoarchitecture and neural connectivity patterns (Barbas and Pandya, 1989; Petrides and Pandya, 2002; Gerbella et al., 2007; Saleem et al., 2014). Such structural heterogeneity suggests functional differentiation within the VLPFC, and indeed, recent functional magnetic resonance imaging (fMRI) studies have revealed functional “patches” within the VLPFC that selectively respond to specific visual categories such as faces, objects, scenes, and colors, and are topographically organized (Tsao et al., 2008; Haile et al., 2019). However, the fine-grained neural circuit basis supporting these functional domains observed at the fMRI scale, particularly the spatial organization of intrinsic connections mediating information processing within and interactions between domains, remains largely unknown.

A fundamental organizational principle for information processing in the cerebral cortex is the concept that functionally related neurons aggregate into columns, forming modules (Mountcastle, 1997). Within the cerebral cortex, connections running largely parallel to the pial surface, often referred to as horizontal or tangential connections, are thought to mediate information transfer between these functional columns (Gilbert and Wiesel, 1979, 1983; Rockland and Lund, 1982). The spatial patterns of these laterally projecting pathways vary across cortical areas; for instance, in the primary visual cortex (V1), a “like-to-like” pattern of such connectivity, where columns with similar functional properties are selectively interconnected, has been reported (Gilbert and Wiesel, 1989; Malach et al., 1993; Bosking et al., 1997). However, it is unclear whether this principle universally applies to higher-order association cortices. For example, our recent study in the inferior temporal cortex (ITC), part of the object recognition pathway, suggested that these types of connections do not necessarily connect sites with similar functional properties selectively (Hu et al., 2025). The organization of such tangential circuits has also been studied in the PFC, the core of higher cognitive functions, where characteristic stripe-like patterns of neuronal labeling, suggestive of organized laterally projecting pathways, have been reported, particularly in the dorsolateral part (DLPFC) (Levitt et al., 1993; Kritzer and Goldman-Rakic, 1995; Pucak et al., 1996). Nevertheless, the detailed spatial organization patterns of these intrinsic connections in other PFC regions remained largely unclear.

Given that the VLPFC differs from the DLPFC in its cytoarchitecture and global connectivity patterns (Petrides and Pandya, 2002; Saleem et al., 2014), the fine-grained spatial distribution pattern of its tangentially organized intrinsic connections, particularly whether regularly structured patterns comparable to the DLPFC stripes exist, had not been fully elucidated. Furthermore, the detailed organization of callosal connections, which support the interhemispheric cooperation required for many higher cognitive functions, was also poorly understood in the PFC (e.g., Schwartz and Goldman-Rakic, 1984). Against this background, the present study investigated the spatial distribution of neurons projecting to microinjection sites of highly sensitive retrograde tracers within the caudal part of the macaque VLPFC (an area considered to correspond mainly to area 45A), analyzing intrinsic connections, contralateral callosal connections, and select extrinsic afferents in detail. As a result, we found that the retrogradely labeled neurons, both ipsilaterally and contralaterally, were not randomly distributed but formed distinct clusters at a demonstrably finer scale than the functional patches observed with fMRI. This finding reveals that both laterally projecting ipsilateral and interhemispheric connections of the VLPFC possess a similar fine-grained architecture characterized by neuronal clustering. This provides crucial anatomical evidence regarding the organization of information processing units within the VLPFC and the structural basis for functional cooperation between the hemispheres.

## 2 Materials and methods

### 2.1 Animals

In this study, we used four adult rhesus macaques (Macaca mulatta; 3 males, 1 female; weight 3.9–10.0 kg). All experimental procedures were conducted in accordance with guidelines for the care and use of laboratory animals established by the National Institutes of Health and were approved by the Zhejiang University Animal Committee (permit number: ZJU20220157). Detailed information about each animal is summarized in Table 1. In all four cases, the same types of retrograde tracers used in this study had also been injected into visual cortical areas V1, V2, and V4 of the contralateral hemisphere for a separate study (Li et al., 2024). We determined that these procedures did not affect the results of the present study because they were located contralaterally and were hierarchically distant from the prefrontal cortex regions where we injected tracers in this study.

**TABLE 1:** Summary of subjects and injection parameters.

Animal and fixation information						Injection information		
Case	Sex	Body weight (kg)	Hemisphere	Survival period after injections (days)	PFA concentration for fixation	AP position	Tracer	Volume (nl)
1	Female	3.9	Left	20	1%	1	CTB-647	50 × 2
						2 (failed)	CTB-488	50 × 2
						3	CTB-555	50 × 2
						4	BDA	40 × 2
2	Male	9.8	Left	21	2%	1	CTB-647	70 × 2
						2	BDA	50 × 2
						3	CTB-555	70 × 2
						4 (failed)	CTB-488	70 × 2
3	Male	10.0	Right	17	2%	1 (failed)	CTB-488	70 × 2
						2	CTB-555	70 × 2
						3	CTB-647	70 × 2
						4	BDA	100 × 2
4	Male	9.5	Left	21	2%	1	BDA	70 × 2
						2	CTB-647	70 × 2
						3	CTB-555	70 × 2
						4	CTB-488	100 × 2

The “AP position” column indicates the sequential order of the four injection sites within each animal, arranged from most anterior (1) to most posterior (4). Injections marked as “failed” indicate cases where no labeled cells were observed in the lateral direction, and the volume is the sum of the two injection depths.

### 2.2 Anesthesia and surgical preparation

Anesthesia was induced with Zoletil (2.5 mg/kg, i.m.) and maintained with 0.5–2.0% isoflurane inhalation during surgery. The animal was fixed in a stereotaxic frame, and a heating pad was used to maintain body temperature at approximately 37°C. Before surgery, atropine (0.10 mg/kg, i.m.) and dexamethasone (0.25 mg/kg, i.m.) were administered to reduce mucus secretion and cerebral edema, respectively. During surgery, vital parameters such as heart rate, body temperature, end-tidal CO_2_, SpO_2_, and respiratory rate were continuously monitored to assess anesthesia depth. Saline and glucose saline were administered intravenously for hydration and energy supply during surgery. Lidocaine was administered subcutaneously at the incision site to reduce pain during scalp incision. After scalp incision, craniotomy was performed on the caudal VLPFC, and the dura was opened to expose relevant anatomical landmarks such as the arcuate sulcus (AS) and principal sulcus (PS). The exposed cortical surface, except for the intended injection site, was covered with artificial dura (Tecoflex, Thermedics Polymer Products) to minimize cortical pulsation.

### 2.3 Tracer injection procedure

Four different types of retrograde tracers were injected into the ventral prearcuate convexity cortex located anterior to the lower arcuate sulcus and ventral to the caudal principal sulcus. Injections were made at four sites parallel to the lower arcuate sulcus, spaced 2.3–4.0 mm apart. According to the average cytoarchitectonic map showing the relationship between sulci and areas in the caudal ventrolateral prefrontal cortex (Gerbella et al., 2007), at least the central 1–2 injection sites were located within area 45A, while the most posterior and anterior sites might have been located in area 45A, or in areas 8r and 12, respectively. However, it should be noted that we could not determine the exact cytoarchitectonic location of each injection site because we did not create sections perpendicular to the cortical surface needed for cytoarchitectonic analysis. Therefore, we indicated the relative position of the injection sites with respect to the major sulci, allowing readers to assess the anatomical location themselves.

The tracers used were four types of retrograde tracers: Alexa Fluor 488-labeled cholera toxin subunit B (CTB-488; Thermo Fisher Scientific, MA), Alexa Fluor 555-labeled CTB (CTB-555; Thermo Fisher Scientific), Alexa Fluor 647-labeled CTB (CTB-647; Thermo Fisher Scientific), and biotinylated dextran amine 3,000 kDa (BDA; Thermo Fisher Scientific). Before injection, the tracers were prepared in 0.1M phosphate-buffered saline at concentrations of 1 mg/ml for CTB variants and 100 mg/ml for BDA.

Tracers were pressure-injected using a Hamilton syringe equipped with a glass pipette (tip diameter: 75–150 μm) mounted on a micromanipulator. Injections were made at a rate of 30–45 nl/min, with a total of 40–100 nl injected per site. The injection pipette was positioned as perpendicular as possible to the cortical surface and initially inserted into the brain to a depth of 2.0 mm. The pipette was then retracted by 0.2 mm for the first injection, and further retracted by 1.0 mm for the second injection. This two-stage injection method created a columnar injection site spanning from the supragranular to infragranular layers. To minimize tracer backflow along the injection track, the pipette was kept in position for 5 min before withdrawal.

### 2.4 Post-surgical care

After tracer injection, the dura was sutured closed, and the original bone flap was replaced, with all gaps sealed with dental cement. The skin incision was then closed with sutures. Buprenorphine (0.005–0.010 mg/kg, i.m.) was administered for postoperative pain relief, and ceftriaxone sodium (25 mg/kg, i.m.) was given prophylactically to prevent infection. The entire surgery, from initial anesthesia to wound closure, took approximately 7–15 h. The animals were closely monitored during recovery from anesthesia and were returned to their home cages only after confirming that their physiological parameters were stable and spontaneous breathing had recovered. For postoperative care, buprenorphine was administered twice daily for 3 days to ensure adequate pain management, and ceftriaxone sodium administration was continued for 7 days after surgery to minimize the risk of infection.

### 2.5 Histological procedures

After a 2–3 week survival period following tracer injections, animals were initially anesthetized with Zoletil (2.5 mg/kg, i.m.), followed by a lethal dose of pentobarbital (50–100 mg/kg, i.v. or i.p.). Deep anesthesia was confirmed by the absence of pupillary reflexes before proceeding with the perfusion. The animals were then perfused transcardially with 0.1M phosphate-buffered saline (PBS), followed by a fixative solution containing 1–2% paraformaldehyde (PFA) and 10% sucrose in phosphate buffer (PB).

Following perfusion, the brain was carefully removed from the skull. The prefrontal cortex from both hemispheres and the ipsilateral inferior temporal cortex were rapidly dissected from the rest of the brain and flattened between glass slides according to previously established methods (Sincich et al., 2003). The flattened cortical tissues were then immersed in 30% sucrose/PB solution and stored overnight at 4°C for cryoprotection. The flattened cortical tissues were then sectioned tangentially at a thickness of 40 μm using a freezing microtome (YAMATO KOKI, Tokyo, Japan). All sections were stored at −20°C in a cryoprotectant solution containing 30% ethylene glycol, 30% glycerol, and 40% 0.1M PBS until further processing. The sections were divided into three series: one series was used for BDA staining, another for cresyl violet (Nissl) staining, and the remaining series was preserved as backup.

For BDA visualization, sections were first rinsed twice in PBS containing 0.3% Triton X-100 (PBST, pH 7.4) to improve permeability. The standard avidin-biotin-peroxidase method (Vectastain ABC kit, Vector Laboratories, Burlingame, CA, USA) was then employed according to the manufacturer’s instructions. Free-floating sections were incubated for 5–10 min in a PBST reaction buffer containing 100 μg/ml 3,3′-diaminobenzidine (DAB; Sigma-Aldrich, St. Louis, MO), 100 μg/ml nickel chloride (for signal enhancement), and 0.01% H_2_O_2_. Between each reaction step, sections were thoroughly rinsed three times with PBST. After the reaction was complete, sections were mounted onto glass slides and coverslipped using an aqueous mounting medium. These sections were also used for the examination of labeling by the fluorescent tracers the fluorescent tracers (CTB-488, CTB-555, and CTB-647).

For cresyl violet counterstaining, sections were first mounted on glass slides and air-dried for several days. The mounted sections were then sequentially rinsed in distilled water, 90% ethanol, and 75% ethanol. Subsequently, sections were stained with 0.1% cresyl violet solution for 5–10 min. Excess cresyl violet was removed by washing the sections in 90% ethanol solution containing 0.8% acetic anhydride for 5–10 min. Following dehydration through ascending concentrations of ethanol, sections were cleared in xylene and coverslipped using a xylene-based mounting medium.

### 2.6 Microscopy and image analysis

Tissue sections were observed through a 10× objective lens using a fluorescence microscope (Olympus, Tokyo, Japan) and digitally photographed with a VS-120 virtual slide scanning system (Olympus, Tokyo, Japan). For fluorescence imaging, appropriate filter sets were used to distinguish different fluorescent dyes. Brightfield imaging was used to visualize BDA-labeled cells and Nissl-stained sections. Digital images were imported into Olyvia to adjust illumination and contrast and then copied to Illustrator 2023 (Adobe Systems, San Jose, CA, USA), where labeled cells were manually identified and plotted. Labeled cells were identified based on their characteristic morphological features and labeling patterns. Specifically, cells showing green, red, and blue fluorescence under appropriate excitation were identified as those labeled with CTB-488, CTB-555, and CTB-647, respectively. BDA-labeled cells were identified by their characteristic brown DAB reaction product and clear cellular morphology.

For laminar classification of labeling, sections containing labeled cells were carefully aligned with adjacent Nissl-stained sections using Photoshop 2023 (Adobe Systems, San Jose, CA, USA), utilizing patterns of blood vessels running perpendicular to the surface. Based on cytoarchitectonic criteria observed in corresponding Nissl-stained sections, labeled cells were classified into three main laminar divisions: supragranular layers (layers 2/3), granular layer (layer 4), and infragranular layers (layers 5/6) (Supplementary Figure 1). It should be noted that precise boundary delineation between layers 2 and 3, and between layers 5 and 6 was technically difficult in tangential sections. However, in tangential sections, the horizontal spread of labeled cells could be more easily confirmed.

Given the close proximity of injection sites for different fluorescent tracers, we also examined the sections for the presence of double-labeled neurons. Throughout our observations, such double-labeled cells were found to be extremely rare, with typically no more than one, if any, observed per section. Due to this scarcity, a systematic quantitative analysis of their distribution was not feasible and was therefore not included in this study.

### 2.7 Quantitative analysis of spatial distribution and neuronal clustering

The x-y coordinate data of labeled cells plotted in Adobe Illustrator were exported for quantitative analysis. These coordinate data were processed using custom Python and MATLAB (MathWorks, Natick, MA, USA) scripts. Pixel coordinates were converted to micrometers (μm) using a user-defined calibration. To visualize the spatial distribution of labeled neurons, density heatmaps were generated by dividing the plotted region into 50 μm × 50 μm square bins and counting the number of neurons within each bin.

For objective identification of clusters, the heatmaps were smoothed with a Gaussian filter (sigma = 400 μm), and local density maxima (peaks) were detected. A contour was drawn at half the maximum height of each peak, and the region enclosed was defined as a putative cluster. To ensure that only robust neuronal aggregates were included in our quantitative analysis, we applied the following criteria. A putative cluster was accepted for measurement if it: (1) could be clearly demarcated by a contour line that did not merge with the dense peri-injection halo; (2) contained a minimum of 25 labeled neurons within its contour in at least one tangential section; and (3) was identifiable across at least three tangential sections. The identity of a single cluster across serial sections was determined based on its position relative to the major sulci and the distinct patterns of radial blood vessels (Supplementary Figure 2).

For each accepted cluster, we performed two types of quantitative measurements. First, for overall size, we selected the single tangential section in which the cluster exhibited its largest cross-sectional area and calculated its major and minor axes using the “regionprops” function in MATLAB. Second, to analyze the laminar distribution, we used adjacent Nissl-stained sections to approximate the boundaries of Layer 4. We then determined for each cluster if the labeled cells were mainly confined to the supragranular layers, the infragranular layers, or spanned both. Finally, to determine adjacent clusters for calculating inter-cluster distances, we employed the Gabriel graphing method (Gabriel and Sokal, 1969; Tanigawa et al., 2005), which defines neighbors based on the empty-circle property. The distances between the peaks of these neighboring clusters were then calculated.

## 3 Results

### 3.1 Injection site placement and verification

**FIGURE 1:**
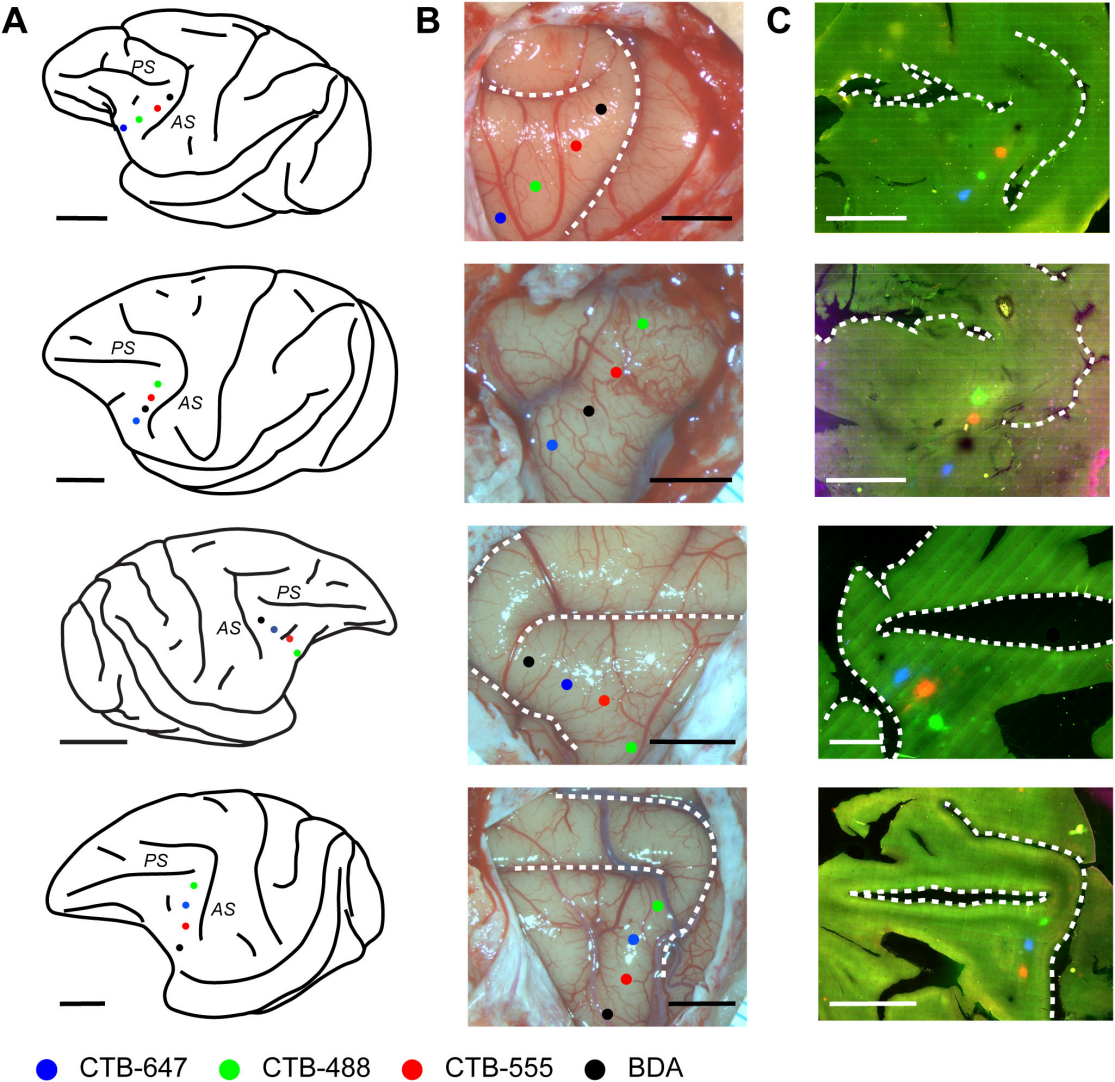
Placement and confirmation of multiple retrograde tracer injections in the macaque VLPFC. **(A)** Schematic lateral views of macaque brains illustrating the approximate VLPFC injection targets. From top to bottom, schematics correspond to Case 1, Case 2, Case 3, and Case 4, respectively. Injections were made into the left hemisphere for Cases 1, 2, and 4, and into the right hemisphere for Case 3. Major sulci in the VLPFC vicinity, the arcuate sulcus (AS) and principal sulcus (PS), are indicated. Injection sites for different tracers are marked with colored dots: CTB-647 (Blue), CTB-488 (Green), CTB-555 (Red), and BDA (Black). **(B)** Corresponding photographs of the exposed prefrontal cortical surface taken at the time of injection, showing the pipette entry points for each tracer, color-coded as in **(A)**. Dashed lines delineate major sulci. **(C)** Fluorescence images of representative tangential sections from each respective case, visualizing the locations of the four tracer injection sites. Scale bars: 1 cm for panels in **(A,C)**; 5 mm for panels in **(B)**.

To investigate the fine-scale structure of neural connections to focal regions within the caudal VLPFC, four distinct retrograde tracers (CTB-488, CTB-555, CTB-647, BDA) were injected into this area in four macaque monkeys (see Table 1). Injections were targeted to the ventral prearcuate convexity cortex, located anterior to the inferior arcuate sulcus and ventral to the caudal tip of the principal sulcus (Figure 1A). Within each animal, the different tracers were administered 2.3–4.0 mm apart. The precise position of each injection relative to cortical sulci was documented using photographs of the exposed cortical surface taken during surgery (Figure 1B). While the tangential sections used in this study precluded a definitive cytoarchitectonic areal classification of the injection sites, we estimated their locations by referencing their relative positions to the major sulci and previously reported cytoarchitectonic and connectional maps (Gerbella et al., 2007; Saleem et al., 2014). Based on this comparison, the two central injection sites were likely located within area 45A, while the most rostral site may have been in Area 12, and the most caudal site in Area 8Av.

**FIGURE 2:**
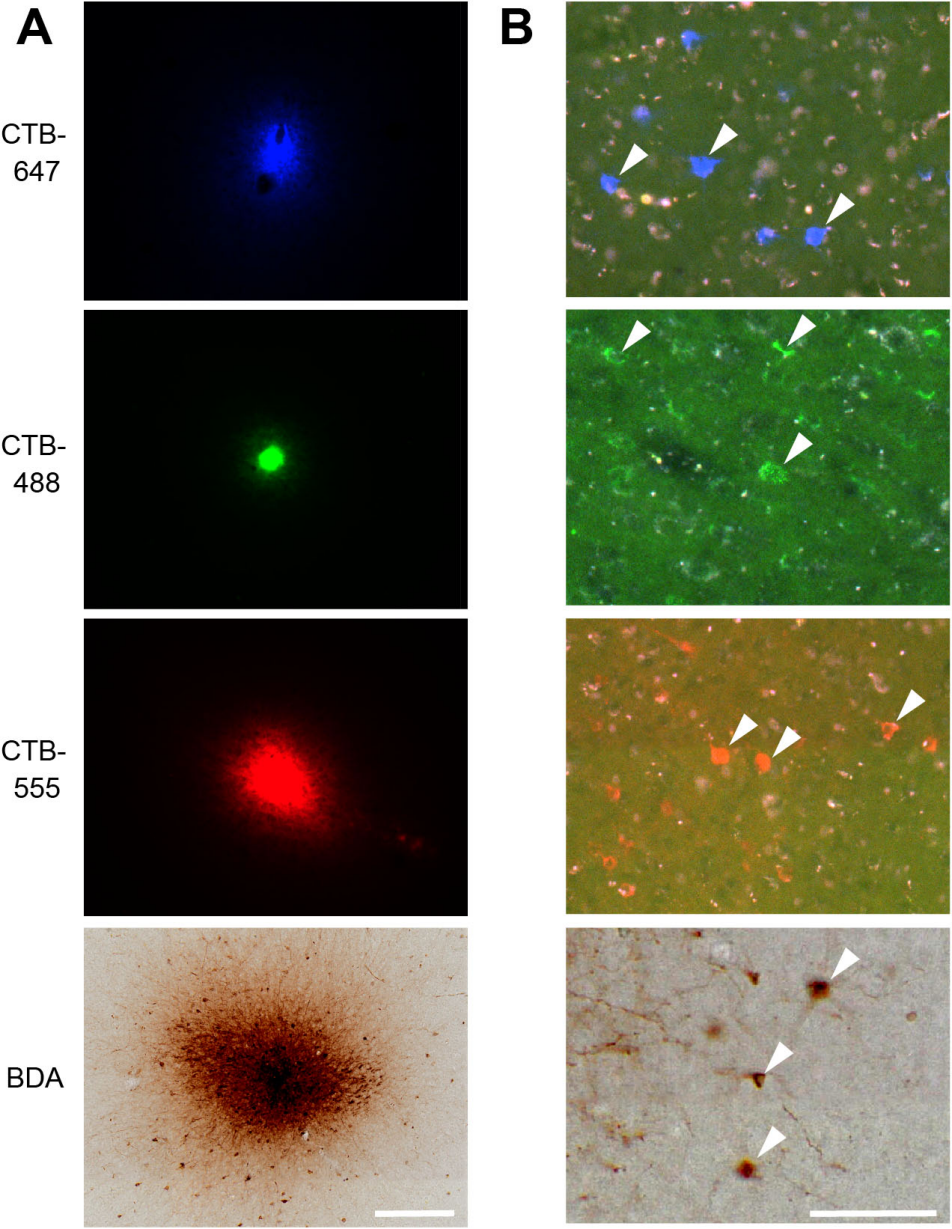
VLPFC injection sites and resulting retrogradely labeled neurons. **(A)** Photomicrographs of four tracer injection sites in Case 1. From top to bottom: CTB-647, CTB-488, CTB-555, and BDA. Fluorescent signals for CTB-647, CTB-488, and CTB-555 are shown under their respective excitation light. The BDA injection site is visualized by its corresponding DAB reaction product under bright-field illumination in the same section. **(B)** High-magnification photomicrographs showing examples of retrogradely labeled neurons (arrowheads) resulting from the corresponding injections shown in **(A)**. Scale bars: 200 μm in **(A)**; 100 μm in **(B)**.

Following perfusion, to facilitate the examination of the extent and patterns of lateral connections to the injection sites, brain blocks containing the VLPFC were carefully flattened, unfolding the sulci (Supplementary Figure 3), and subsequently sectioned tangentially. This procedure yielded tangential sections encompassing a wide area of the VLPFC, including cortex previously situated within sulci. Subsequent histological processing enabled the visualization of both the injection sites and the resulting labeled neurons within these sections (Figures 1C, 2). Of the 16 total injections performed across all animals, three were deemed unsuccessful due to the absence of labeled neurons or labeling strictly confined to the injection core. Detailed analysis of labeled neuron distributions was therefore conducted on the remaining 13 successful injections. The tangential size of the successful injection sites ranged from approximately 60 to 250 μm. Examination of Nissl-stained tangential sections confirmed that these successful injections effectively spanned multiple cortical layers, including both supragranular and infragranular layers (Supplementary Figure 1).

### 3.2 General organization: widespread clustered connectivity from VLPFC

**FIGURE 3:**
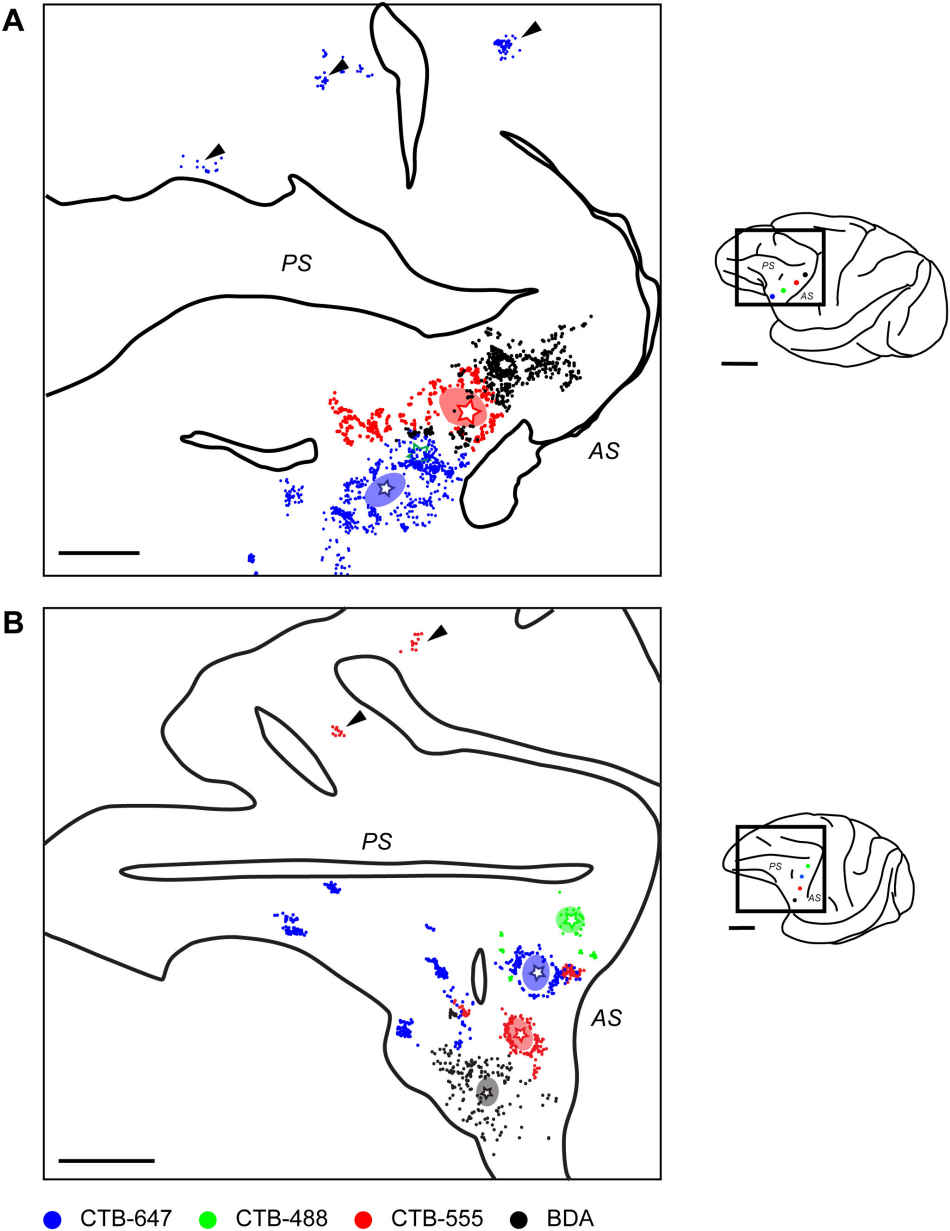
Distribution of ipsilaterally projecting neurons in two representative cases. **(A)** Reconstructed map from a flattened tangential section of the ipsilateral prefrontal cortex in Case 1, illustrating the distribution of retrogradely labeled neurons originating from different VLPFC injection sites (stars; color-coded as in Figure 1). Arrowheads indicate clusters observed in the distal DLPFC. **(B)** A similar reconstructed map from another representative case (Case 4). Conventions are consistent across both panels. Ovals surrounding the injection sites represent the peri-injection halos. Individual colored dots correspond to labeled neurons (Blue: CTB-647; Green: CTB-488; Red: CTB-555; Black: BDA). Solid lines indicate the unfolded positions of major sulci, the principal sulcus (PS) and arcuate sulcus (AS). In Case 1, neurons labeled by CTB-488 (Green) were not observed, likely due to a failed injection. For each panel, the schematic on the right shows a lateral view of the brain, with a rectangle indicating the approximate location of the mapped region. Scale bars: 5 mm for the left and 1 cm for the right.

**FIGURE 4:**
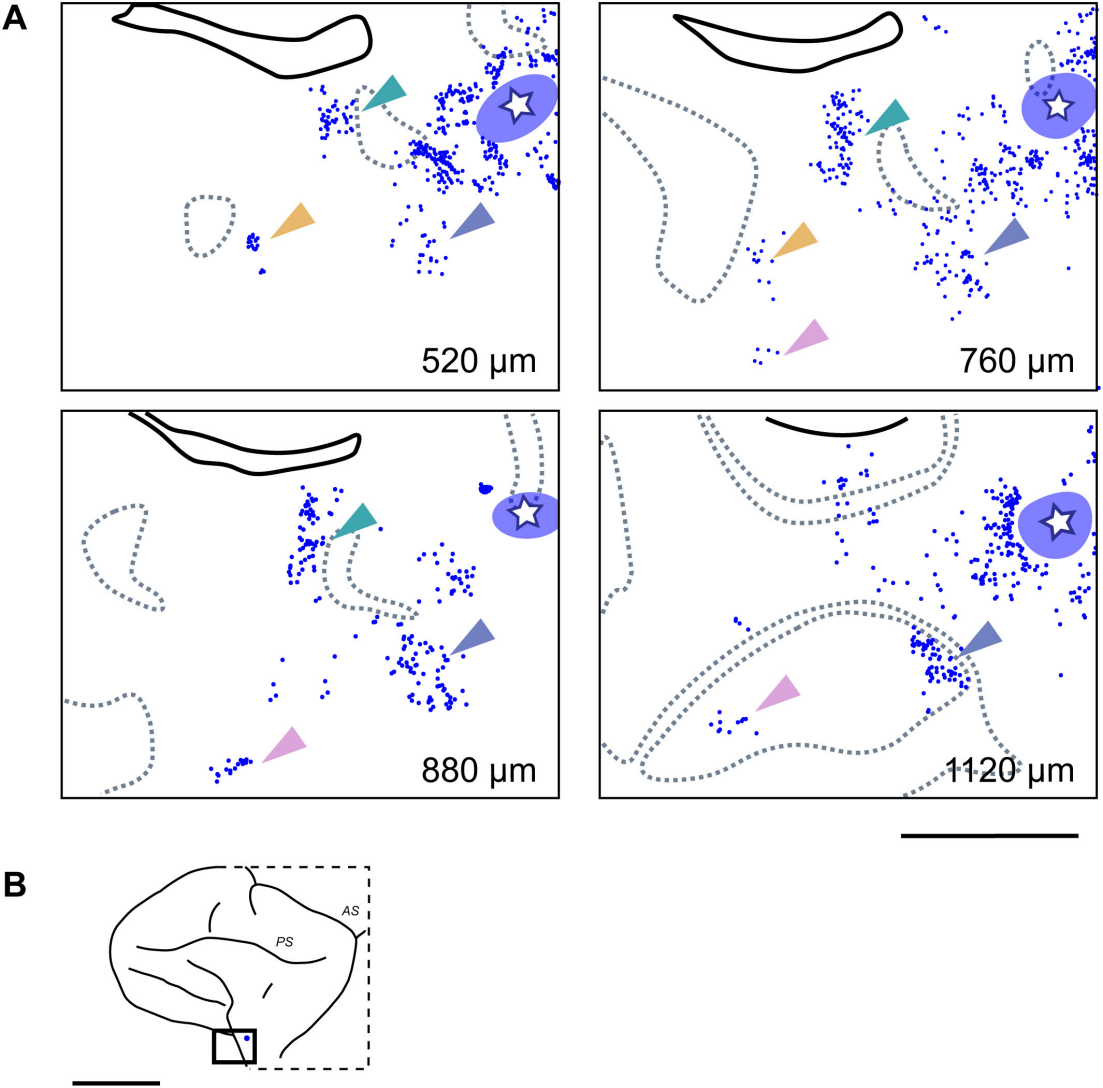
Clustered ipsilateral connections at different cortical depths in the VLPFC. **(A)** Retrogradely labeled neurons (blue dots; from CTB-647 injection, Case 1, Position 1) forming distinct clusters in tangential sections at four different depths from the pial surface. The white star within the translucent blue oval indicates the injection site and its surrounding halo, respectively. Dotted gray lines indicate the approximate upper and lower boundaries of Layer 4, based on adjacent Nissl-stained sections. Arrowheads of different colors highlight representative individual neuronal clusters that maintain a consistent relative position across different depths. **(B)** Schematic lateral view of the macaque brain, with a rectangle indicating the approximate location of the magnified region shown in **(A)**. Scale bars: 5 mm in **(A)**; 1 cm in **(B)**.

By mapping the locations of neurons labeled by each tracer on tangential sections containing the injection site, 2D distribution plots were obtained. Within these plots, a dense aggregate of labeled neurons was observed within a radius of approximately 0.5–1.0 mm around the core of the injection site (Figure 3), indicating the presence of strong local lateral connections (often called “halos”) within this VLPFC region. In the region beyond this immediate halo, labeled neurons were not uniformly distributed, but formed distinct and spatially separated clusters (Figure 3, arrowheads). Such clusters that appeared laterally were observed up to a distance of 12.5 ± 5.8 mm (mean ± SD, *n* = 13) from the injection site center. These aggregates were not incidental two-dimensional formations, but consistently maintained their tangential position across serial sections sampling different cortical depths (Figure 4), suggesting they form radially oriented, columnar-like structures. We estimated the areal locations of these clusters by referencing their positions relative to the major sulci against previously reported cytoarchitectonic and connectional maps (Gerbella et al., 2007; Saleem et al., 2014). Within the ipsilateral PFC, clusters were widely distributed; in the VLPFC, they were found in putative Areas 45A, 46v, 12l, and 8Av, while in the DLPFC, they were seen in Areas 46d and 8B. This overall distribution was largely consistent with previous reports (Gerbella et al., 2010).

**FIGURE 5:**
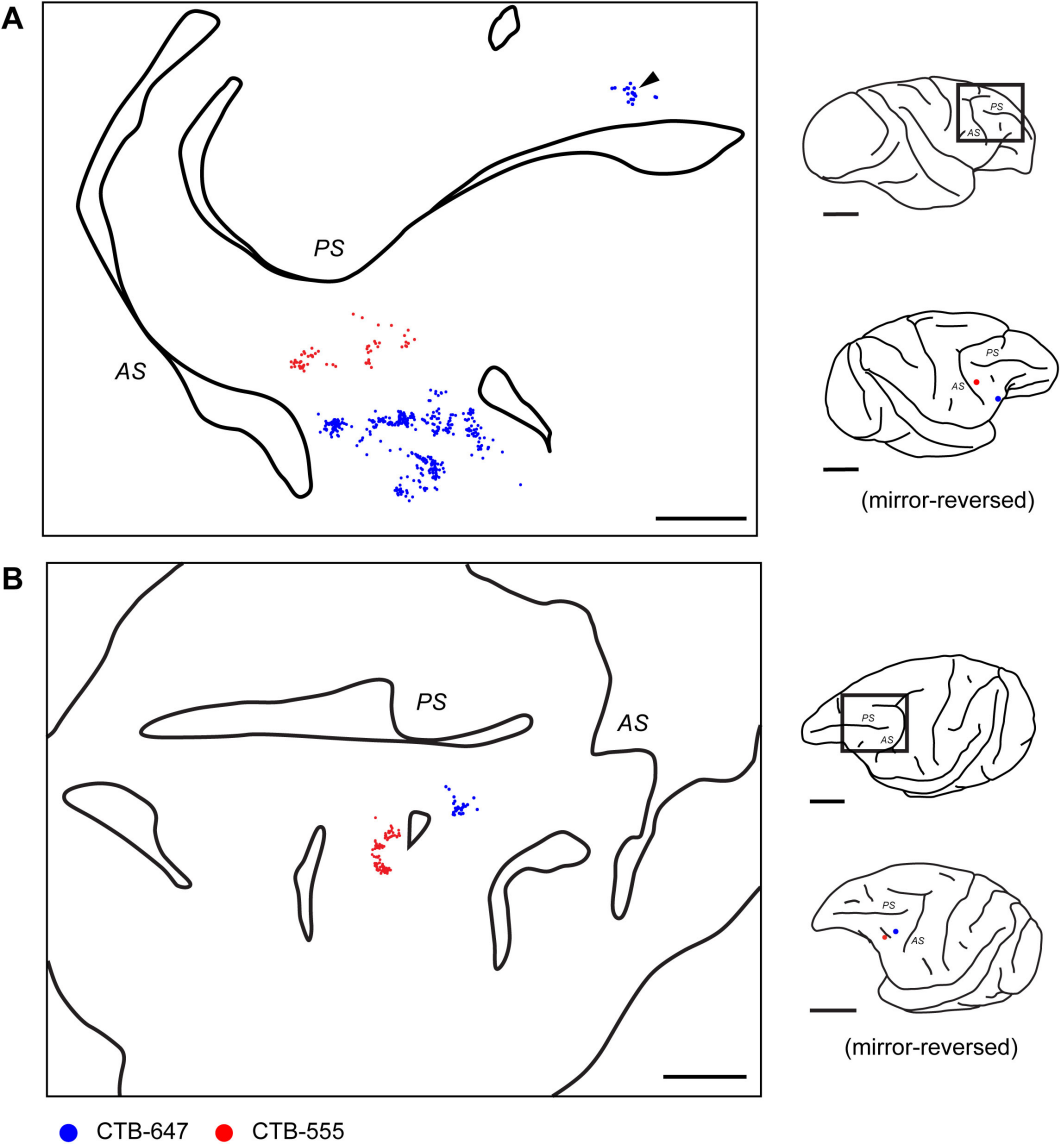
Distribution of contralateral neurons projecting to caudal VLPFC. **(A)** Reconstructed 2D map from a flattened tangential section of the contralateral prefrontal cortex in Case 1, illustrating the distribution of retrogradely labeled neurons originating from two VLPFC injections (CTB-647, blue dots, position 1; CTB-555, red dots, position 3) in the opposite hemisphere. Arrowhead indicates a cluster observed in the distal DLPFC. **(B)** A similar reconstructed map from another representative case (Case 3, Position 2 and 3). Conventions for depicting labeled neurons and sulci (PS and AS) are similar for both panels. For each panel, the schematics on the right show a lateral view of the brain with a rectangle indicating the mapped region, and below it, the locations of the tracer injections in the source hemisphere. Note that the schematic of the injected hemisphere has been mirror-reversed for ease of comparison with the contralateral map. Scale bars: 5 mm for the left and 1 cm for the right.

**FIGURE 6:**
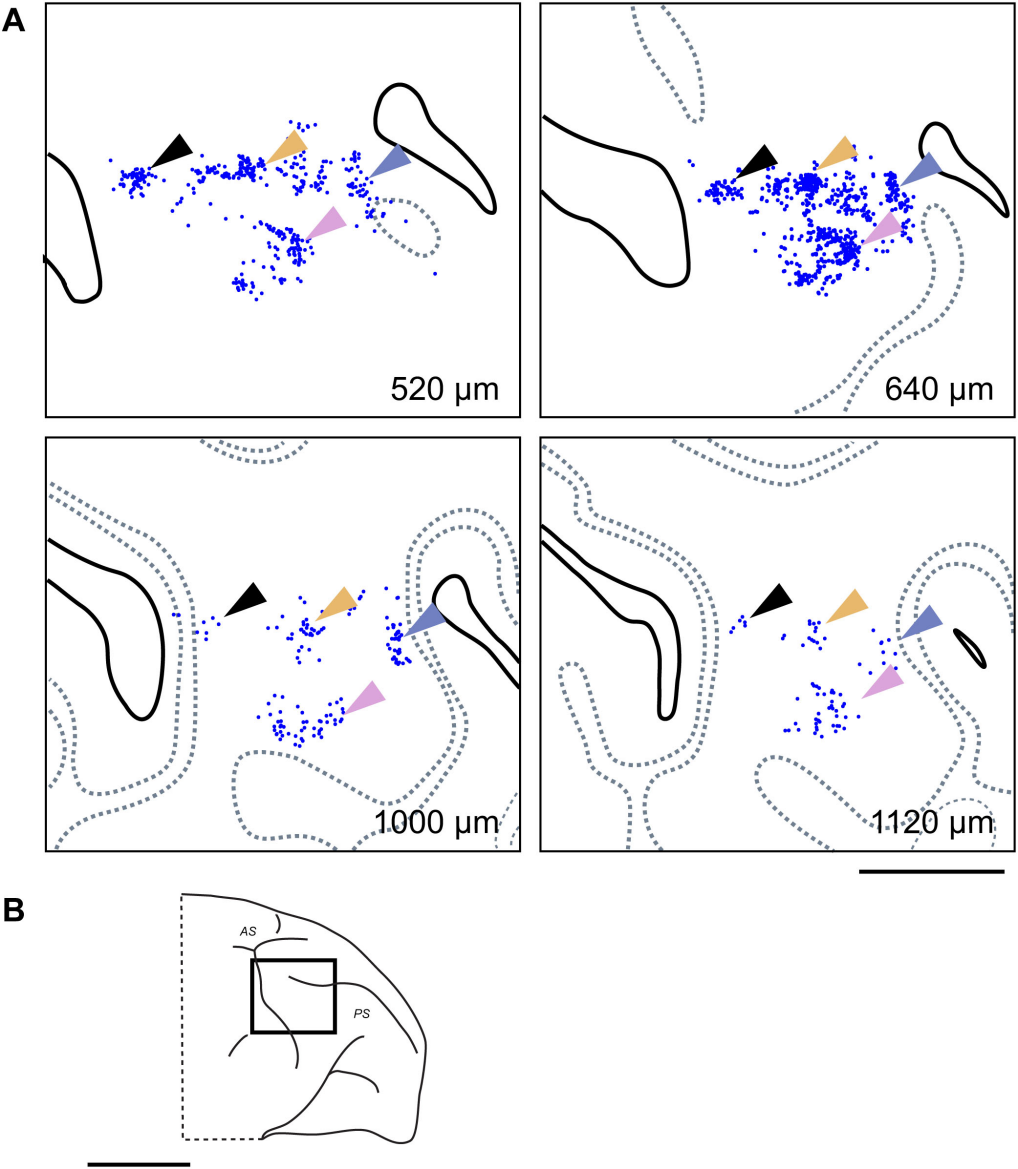
Clustered contralateral connections at different cortical depths. **(A)** Examples of clusters formed by CTB-647 labeled neurons (blue dots) observed in tangential sections at four different depths in the contralateral VLPFC of Case 1 (Position 1). Arrows of different colors highlight representative neuronal clusters that maintain a consistent relative tangential position across these depths, demonstrating a clustered distribution pattern and suggesting a columnar-like organization for these callosal inputs. Conventions are similar to those in Figure 4A. **(B)** Schematic lateral view of the macaque brain, with a rectangle indicating the approximate location of the mapped region in **(A)**. Scale bars: 5 mm in **(A)**; 1 cm in **(B)**.

In addition to ipsilateral connections, several injections resulted in retrogradely labeled neurons in the contralateral hemisphere, primarily located within the VLPFC region homotopic to the injection sites (Figure 5). The distribution of these callosally projecting neurons also exhibited a distinct tendency to form clusters. Similar to the ipsilateral findings, these contralateral clusters were often observed across multiple tangential sections representing different cortical depths (Figure 6). In some instances where multiple, distinct injections in the same animal successfully labeled contralateral neuron groups, the relative spatial arrangement of clusters labeled by different tracers appeared to correspond with the relative positions of the injection sites themselves (Figures 5A, B, lower right in each), indicating a degree of topographical organization in these interhemispheric projections. Although fewer in number, these contralateral clusters exhibited a similar areal distribution pattern to that seen ipsilaterally (Figure 5).

### 3.3 Quantitative characterization of connectional clusters

Based on our qualitative observations that connectional clusters were most consistently and prominently observed in the supragranular layers, we focused our main quantitative analysis on this compartment. Therefore, unless otherwise specified (see Section “3.6 Clustered organization of projections from STS to VLPFC” for a detailed laminar analysis), all cluster properties reported in the following sections were measured from clusters identified within the supragranular layers (Layers 2/3).

**FIGURE 7:**
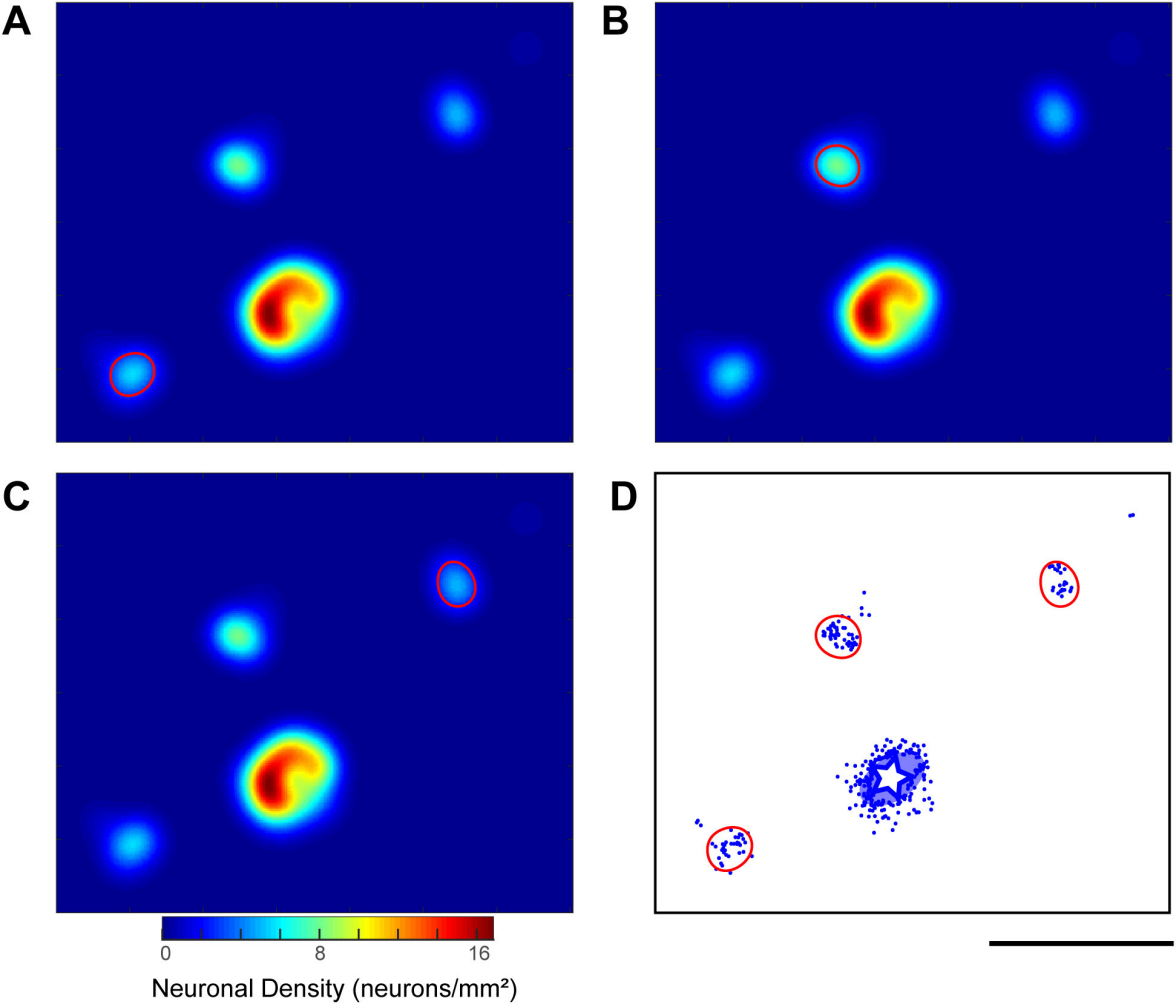
Quantitative analysis of labeled neuron distribution and identification of neuronal clusters. **(A–C)** Density heatmaps illustrating the process of cluster identification, using a representative example from a CTB-647 injection in the ipsilateral hemisphere of Case 2 (Position 1). These heatmaps are derived from a tangential section at an approximate depth of 760 μm. The color bar indicates neuronal density (neurons/mm^2^). In each panel **(A,B,C)**, a contour line (demarcating a single cluster) is drawn at half the height of a distinct local density peak identified on the smoothed heatmap (see “2 Materials and methods” for details of smoothing and peak detection). **(D)** Visualization of all identified cluster boundaries (red contours), established using the method exemplified in **(A–C)**, superimposed on the corresponding raw neuron distribution plot (blue dots) from the same tangential section. The white star and the surrounding translucent blue oval indicate the injection site and its surrounding halo, respectively. Scale bar: 5 mm (applies to all panels).

To quantitatively characterize the observed cluster structure, we first created density heatmaps of labeled neurons from the plotted coordinates to visually confirm their non-uniform spatial distribution (Figures 7A–C). Subsequently, we objectively defined the boundaries of neuronal clusters by identifying local density peaks and drawing contour lines corresponding to half the peak density value (Figure 7D; see “2 Materials and methods” for details). This method allowed us to measure and compare the properties of clusters across different hemispheres and cortical regions.

**FIGURE 8:**
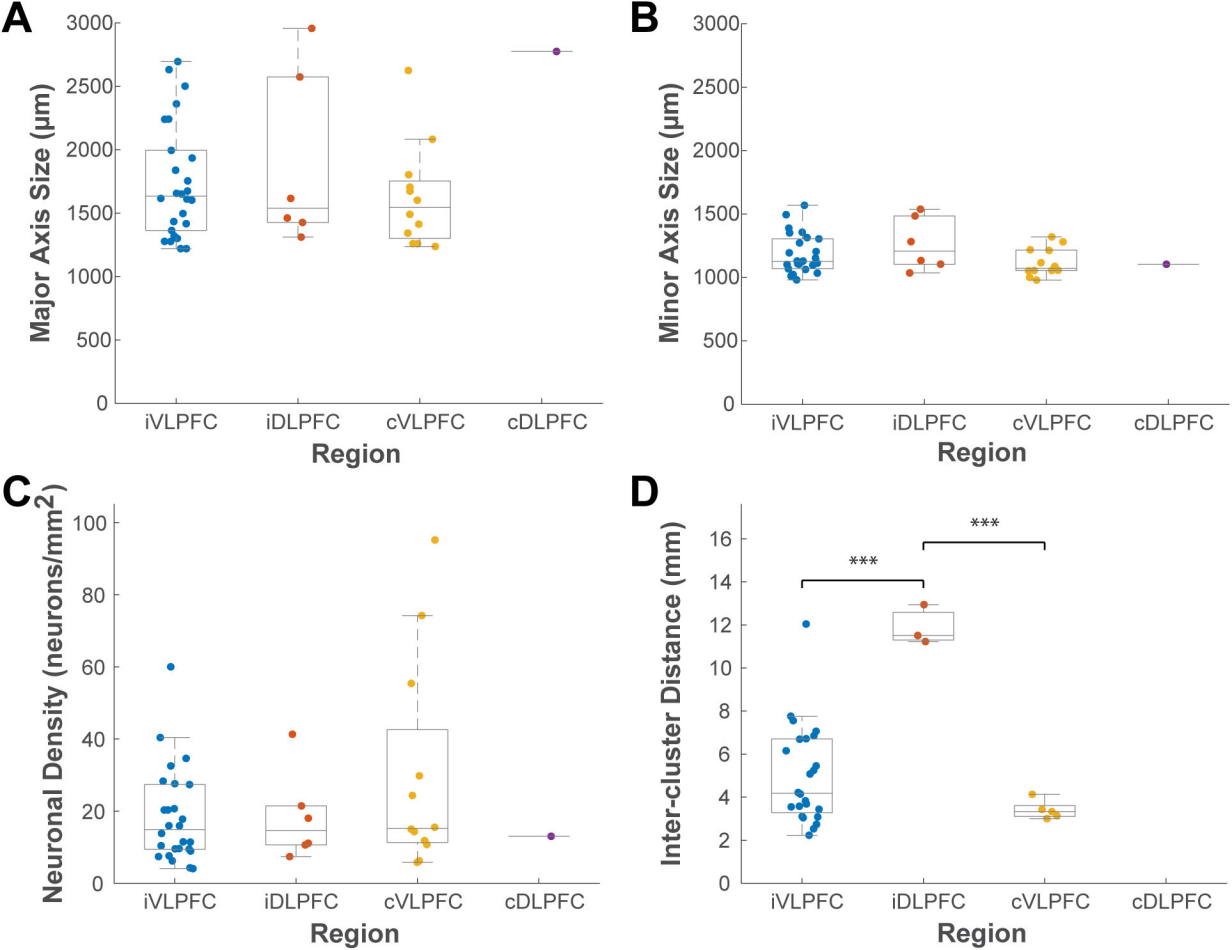
Box plots comparing the distributions of cluster properties across four cortical locations. **(A)** major axis size, **(B)** minor axis size, **(C)** neuronal density, and **(D)** inter-cluster distance. Data are grouped by location: ipsilateral VLPFC (iVLPFC), ipsilateral DLPFC (iDLPFC), contralateral VLPFC (cVLPFC), and contralateral DLPFC (cDLPFC). Each colored dot represents a single cluster. On each box plot, the central line indicates the median, the box edges represent the 25th and 75th percentiles (the interquartile range), and the whiskers extend to the most extreme data points. Asterisks indicate statistically significant differences between groups as determined by one-way ANOVA with *post hoc* Tukey’s HSD tests (*P < 0.05; **P < 0.01; ***P < 0.001). The number of clusters analyzed for each group is detailed in the “3 Results” section.

This analysis provided a detailed quantitative description of the clusters (Figure 8). In the ipsilateral hemisphere, the 26 VLPFC clusters had average dimensions along the major and minor axes of 1,744 ± 443 μm and 1,180 ± 152 μm, respectively, while the 6 DLPFC clusters measured 1,891 ± 634 μm and 1,263 ± 190 μm. In the contralateral hemisphere, the 12 VLPFC clusters had average dimensions of 1,625 ± 388 μm and 1,119 ± 106 μm. The average inter-cluster distance was 5.0 ± 2.2 mm in the ipsilateral VLPFC, 3.4 ± 0.4 mm in the contralateral VLPFC, and was largest in the ipsilateral DLPFC at 11.9 ± 0.8 mm.

A one-way ANOVA was performed to test for differences among these groups. This analysis revealed that the most significant difference was in their spatial arrangement, not their size. The inter-cluster distance varied significantly among the groups (*P* < 0.001, *n* = 32). *Post-hoc* tests indicated that the inter-cluster distance in the ipsilateral DLPFC was significantly greater than that in both the ipsilateral VLPFC (Tukey HSD, *P* < 0.001) and contralateral VLPFC (*P* < 0.001), although this may simply reflect the small number (*n* = 6) of sparsely distributed clusters identified in the ipsilateral DLPFC. In contrast, no significant differences among the groups were found for any measure of cluster size (major axis, *P* = 0.130; minor axis, *P* = 0.297; *n* = 45) or for neuronal density (*P* = 0.334, *n* = 45). This suggests that the size and density of these connectional units may be a conserved feature.

**FIGURE 9:**
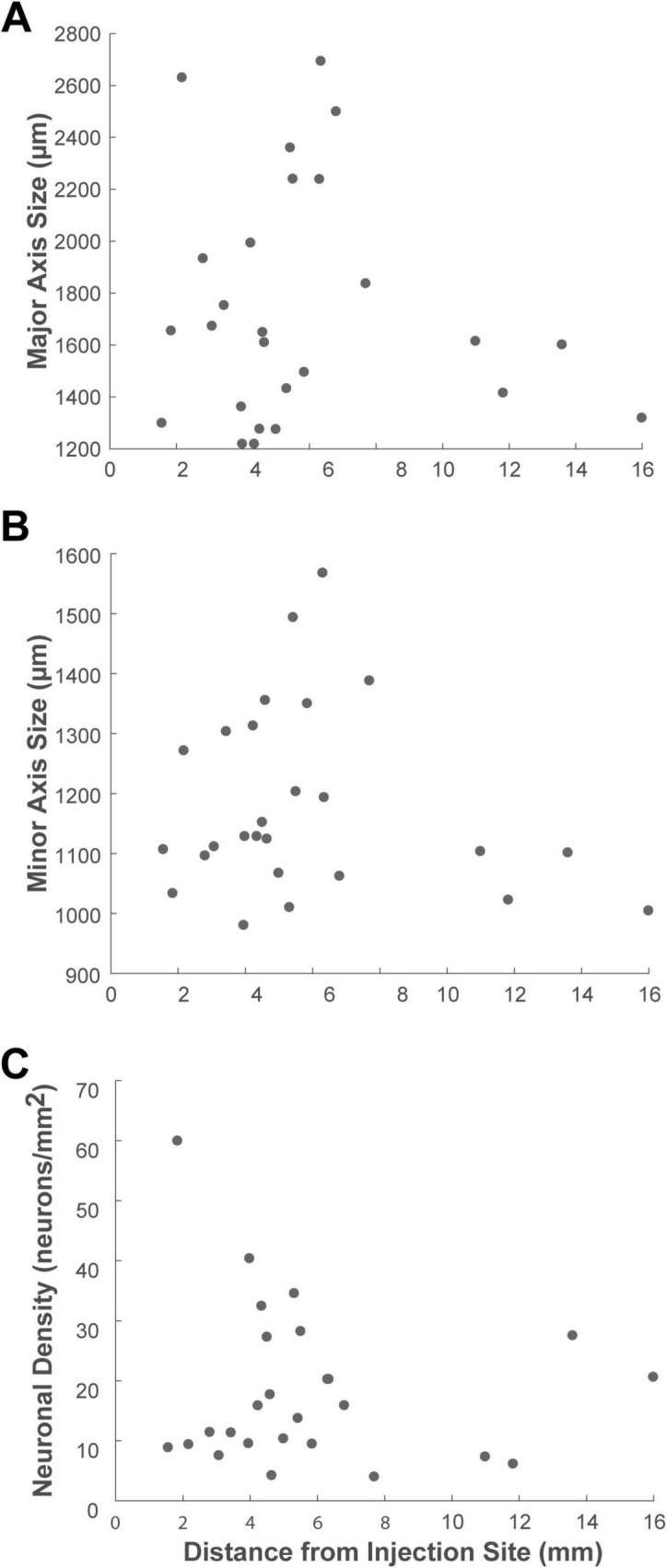
Scatter plots showing the relationship between cluster properties and the distance from the injection site. The analysis illustrates the correlation between the distance of each cluster from the injection site center and its **(A)** major axis length, **(B)** minor axis size, and **(C)** neuronal density. Each dot represents a single cluster identified in the ipsilateral VLPFC.

Finally, to examine if cluster characteristics changed as a function of their distance from the injection site within the VLPFC, we performed a correlation analysis. This revealed no significant correlation between distance and any of the cluster metrics (Pearson’s correlation, *P* > 0.4 for all) (Figure 9). This finding suggests that the principles governing these cluster properties are independent of the physical connection distance.

### 3.4 Influence of injection anteroposterior position on cluster characteristics

Within each animal, our four injection sites were positioned sequentially from anterior to posterior, roughly following the curve of the inferior arcuate sulcus (Figure 1). While the precise locations relative to sulcal landmarks were not stereotyped across animals, we tested if cluster properties were influenced by their anteroposterior (AP) position. Several observations suggested that the AP position of injections influenced cluster distribution. First, retrograde labeling from the most posterior (4th) injection site was limited; while two of the four such injections produced labeled cells, they were confined to the local halo and failed to form distinct distal clusters. Second, all clusters identified in the DLPFC (six ipsilateral and one contralateral) originated exclusively from the 1st and 2nd injection sites from the anterior.

**FIGURE 10:**
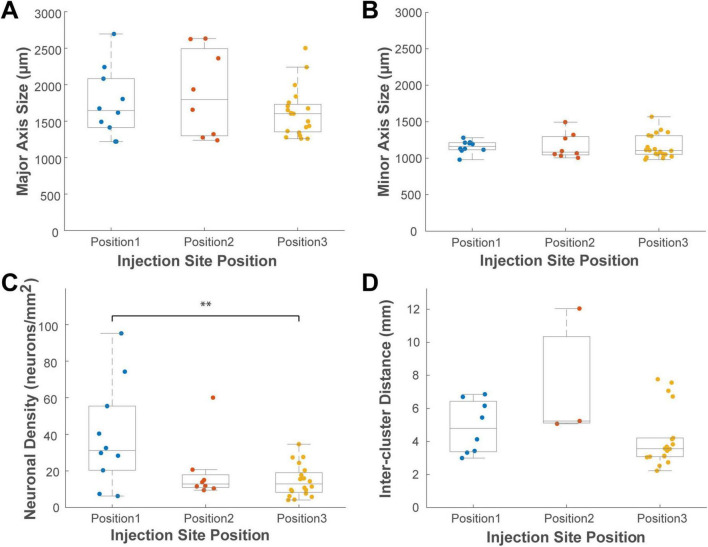
Box plots illustrating the effect of anteroposterior (AP) injection position on the properties of VLPFC clusters of both hemispheres. Panels show the distributions of **(A)** major axis size, **(B)** minor axis size, **(C)** neuronal density, and **(D)** Inter-cluster distance. Data are grouped by the three most anterior injection positions. Each colored dot represents a single cluster from either the ipsilateral or contralateral hemisphere. Conventions for the box plots are as described for Figure 8. Asterisks indicate a significant main effect of AP position as determined by two-way ANOVA, with significant pairwise differences identified by *post hoc* Tukey’s HSD tests (*P* < 0.05).

To assess the influence of injection position while controlling for hemisphere, we performed a two-way ANOVA on the characteristics of ventral PFC clusters, with hemisphere (ipsilateral vs. contralateral) and AP injection position (1st, 2nd, and 3rd) as factors (Figure 10). This analysis revealed a significant main effect of AP position on neuronal density (Figure 10C; *P* = 0.003, *n* = 38). A *post-hoc* test showed that clusters originating from the most anterior injection site (position 1) were significantly denser than those from the third site (Tukey HSD, *P* = 0.002). No other significant main effects or interaction effects on any cluster property were observed (*P* > 0.05 for all). Taken together, these results suggest that the location of the target within the caudal VLPFC influences both the cluster characteristics and the broader spatial distribution of its projecting neurons.

### 3.5 Laminar profile of connectional clusters

To investigate the three-dimensional organization of the connectional architecture, we analyzed the laminar distribution of all identified clusters. Using the approximate boundary of layer 4 as a landmark (see “2 Materials and methods”), each cluster was classified as being confined to the supragranular layers, confined to the infragranular layers, or spanning both compartments. Of the 45 clusters identified in the prefrontal cortex of both hemispheres, the majority (31 clusters, or 69%) were found to span both the supragranular and infragranular layers. This trans-laminar organization was prominent in the ipsilateral VLPFC, where 85% of clusters (22 of 26) spanned both layers, with the remaining four clusters confined to the supragranular layers. In the ipsilateral DLPFC, half of the clusters (3 of 6) spanned both layers, while the other half were restricted to the supragranular layers. A similar prevalence was observed in the contralateral hemisphere; of the 12 VLPFC clusters, five (42%) were trans-laminar, four were supragranular-only, and three were infragranular-only. The single cluster identified in the contralateral DLPFC also spanned both layers.

To further quantify the columnar properties of these clusters, we examined their metrics at different cortical depths. Since the thickness of the flattened cortical tissue varied depending on its original location relative to sulci, we used the position of layer 4 as a standardized reference for depth, rather than the distance from the pial surface. In cases where an adjacent Nissl-stained section was available, the depth of the section containing the center of Layer 4 was defined as the zero reference point (0 μm). However, due to the discontinuous sampling of our Nissl series (one in every three sections), the precise location of Layer 4 could not always be directly visualized for every cluster-containing section. In these instances, we estimated the zero depth reference based on the characteristic extremely low density of labeled projection neurons in Layer 4 compared to the supragranular and infragranular layers (Tanigawa et al., 1998). It should be noted that the total cortical thickness observed in our tangential sections is reduced compared to the *in vivo* state. This is a known consequence of the physical flat-mounting procedure, which involves compressing the tissue under weight during postfixation (Sincich et al., 2003). To standardize the analysis of clusters observed at various distances from layer 4, we binned the data into 100 μm intervals and calculated the average of relevant parameters within each bin.

**FIGURE 11:**
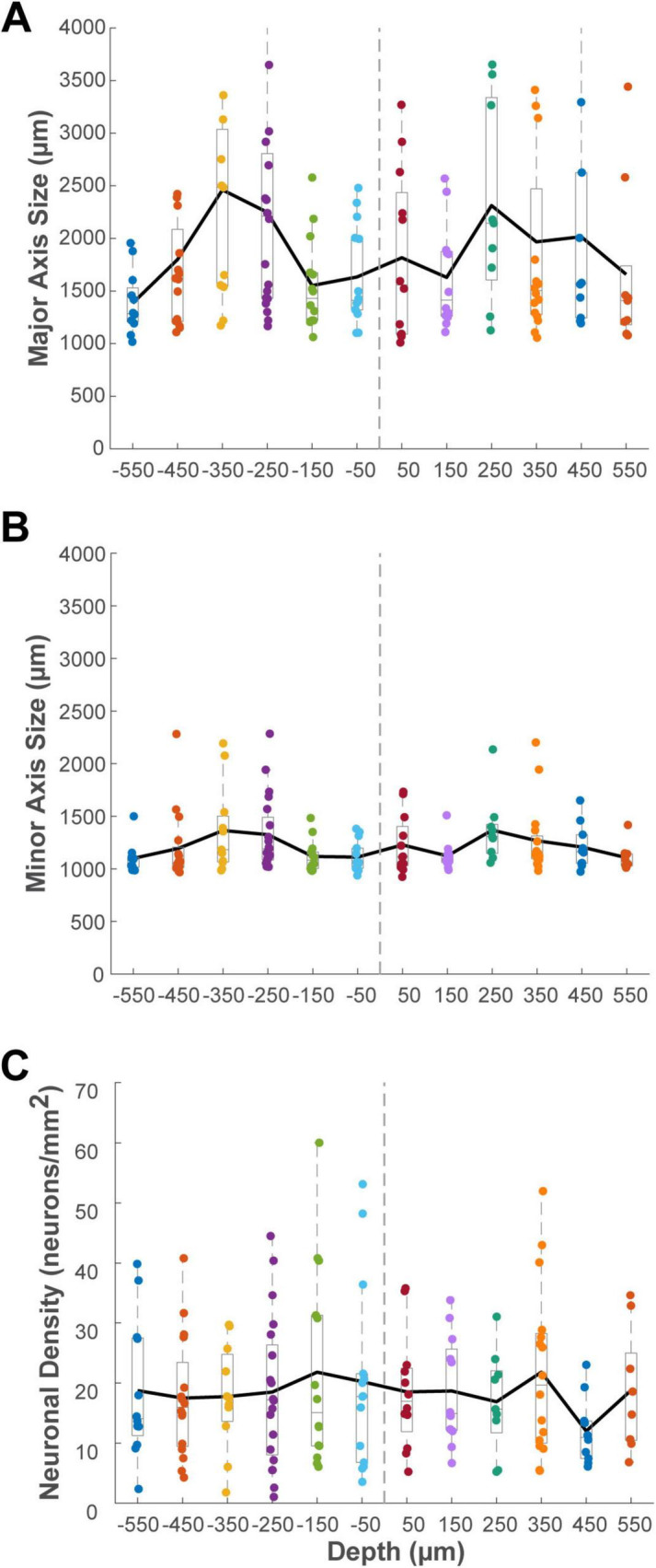
Histograms showing the distribution of cluster metrics across different cortical depths relative to layer 4 (0 μm) for identified clusters in the ipsilateral VLPFC. Sections located in the supragranular compartment were assigned negative depth values, while those in the infragranular compartment were assigned positive values. Data are binned in 100 μm intervals. **(A)** Average major axis size (μm). **(B)** Average minor axis size (μm). **(C)** Average neuronal density (neurons/mm^2^). The solid black line in each panel connects the mean value for each depth bin. Conventions for the box plots are as described for Figure 8.

This analysis was performed on 22 clusters in the ipsilateral VLPFC (Figure 11), 3 in the ipsilateral DLPFC, 5 in the contralateral VLPFC, and 1 in the contralateral DLPFC (Supplementary Figure 4), excluding those with incomplete depth information. Histograms of cluster metrics (major axis size, minor axis size, and density) were created for each depth bin within each region. To compare metrics between the supragranular and infragranular portions of each cluster, we averaged the values from all sections belonging to each compartment for a given cluster. Two-tailed paired *t*-tests revealed no significant differences between the supragranular and infragranular layers for any metric in any region (major axis size, minor axis size, neuronal density, all *P* > 0.05). We also performed a two-way ANOVA to evaluate the effect of injection AP position on the laminar profile of clusters in both the ipsilateral and contralateral VLPFC, respectively. This analysis, similar to the analysis in Section “3.4 Influence of injection anteroposterior position on cluster characteristics,” revealed a significant main effect of anteroposterior injection position on neuronal density in the ipsilateral VLPFC (*P* = 0.005, *n* = 44). *Post-hoc* tests showed that clusters originating from the anterior-most injection site (position 1) were significantly denser than those from position 3 (Tukey HSD, *P* = 0.004). However, no significant main effect of layer (supragranular vs. infragranular) or any interaction effect was observed (all *P* > 0.05). These results demonstrate that the connectional clusters in the PFC are predominantly trans-laminar structures that maintain consistent size and density across their vertical extent. This trans-laminar organization, observed for the majority of connectional clusters, provides anatomical evidence for a columnar-like architecture underlying these long-range connections.

### 3.6 Clustered organization of projections from STS to VLPFC

**FIGURE 12:**
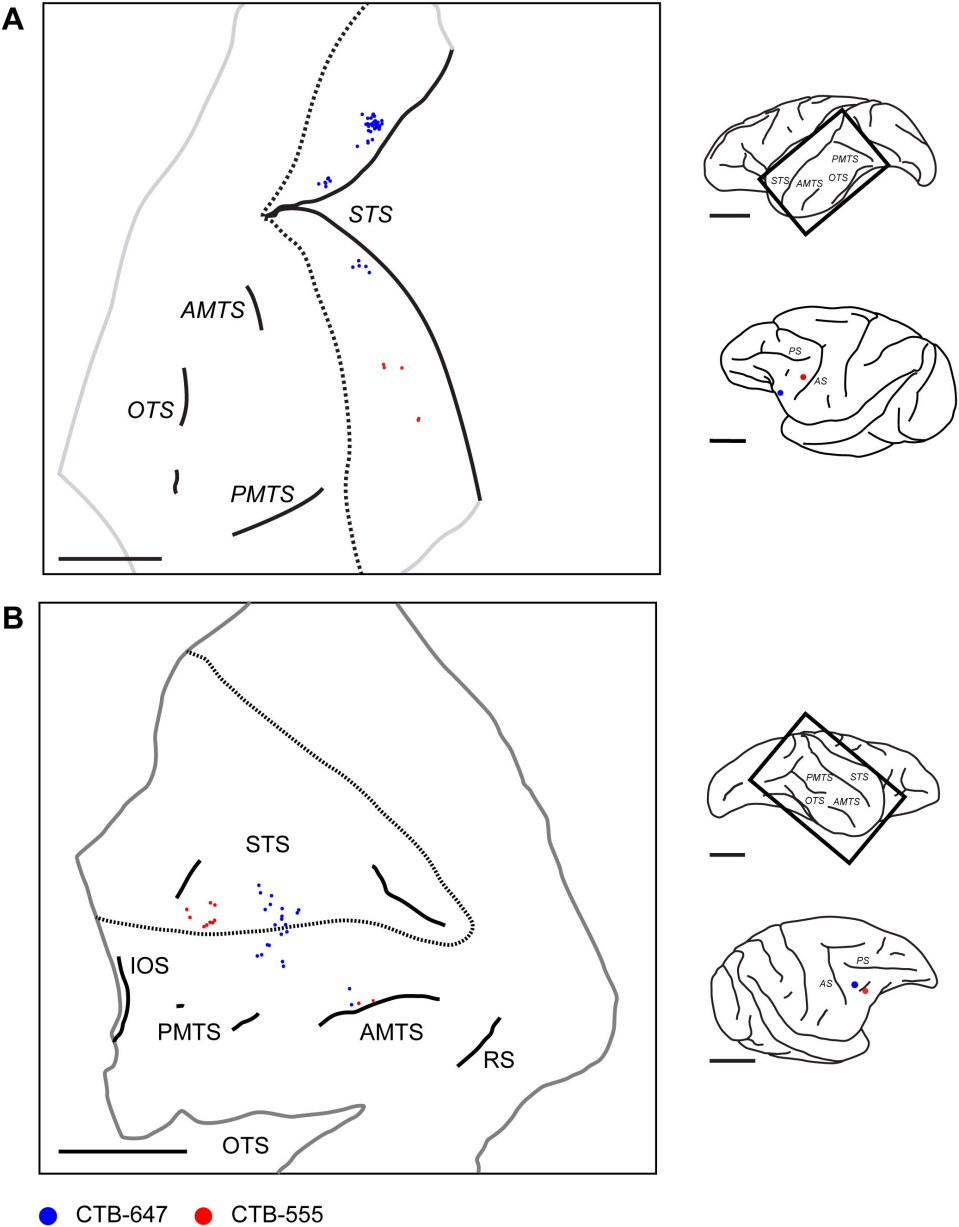
Distribution of retrogradely labeled neurons in the ipsilateral temporal lobe projecting to VLPFC. **(A)** Reconstructed 2D map from a flattened tangential section of the ipsilateral temporal lobe in Case 1 (Position 1 and 3), illustrating the distribution of retrogradely labeled neurons (Blue: CTB-647; Red: CTB-555) originating from two VLPFC injections in the ipsilateral hemisphere. **(B)** A similar reconstructed map from Case 3 (Position 2 and 3). Conventions are consistent across both panels. For each panel, the schematics on the right show a lateral view of the brain with a rectangle indicating the approximate location of the mapped region, and a separate view showing the injection sites in the injected ipsilateral PFC. Dotted lines indicate the approximate dorsal and ventral lips of the STS. Trimmed edges of the sections are shown with gray line. The approximate depths of two sections from the pial surface are 1,400 μm and 1,480 μm for **(A,B),** respectively. STS, superior temporal sulcus; OTS, occipital temporal sulcus; AMTS, anterior middle temporal sulcus; PMTS, posterior middle temporal sulcus. IOS, inferior occipital sulcus; RS, rhinal sulcus. Scale bars: 1 cm for all panels.

Beyond intra-PFC cortico-cortical connections, our investigation extended to afferent inputs from the superior temporal sulcus (STS). Following retrograde tracer injections into the caudal VLPFC, labeled neurons were identified in the ipsilateral temporal lobe. These afferent projection neurons were located predominantly in the anterior STS, from its fundus and banks to the cortex on its ventral lip, corresponding to putative Areas STSv/f and TEad (Kravitz et al., 2013; Figure 12). Examination of tangential sections revealed that these labeled projection neurons were not uniformly distributed but instead exhibited a distinct clustered spatial organization, typically appearing as aggregates with diameters of approximately 500–1,500 μm. Furthermore, we observed a topographical tendency within this temporo-prefrontal projection. In one case, for instance, a more anterior VLPFC injection (position 1) resulted in more dorsal labeling within the STS, whereas a more caudal injection (position 3) led to more ventral labeling (Figure 12A). Similarly, in another case, two injections presumed to be in area 45A (positions 2 and 3) resulted in labeled cells distributed from the ventral part of the anterior STS, across its ventral lip, and into the adjacent ventral cortex (Figure 12B).

We also analyzed the laminar distribution of these labeled neurons within the STS. Calculating the total number of neurons across 18 sections from four injection sites in two cases, our analysis revealed a strong bias for the superficial layers, with a total of 610 labeled neurons found in the supragranular layers compared to 180 in the infragranular layers. However, as these STS clusters did not consistently meet all of our predefined quantitative criteria for cluster measurement, a detailed quantitative analysis of their size was not performed. Nonetheless, the qualitative observation of this clustered arrangement suggests that the projection from the STS to the VLPFC possesses an organized architecture, analogous to the patterns observed for connections within the PFC itself.

## 4 Discussion

This study provides anatomical evidence for a fine-scale clustered architecture of connections originating from the macaque caudal VLPFC. Our retrograde tracer injections were centered in the caudal VLPFC, a region largely corresponding to area 45A, with potential involvement of adjacent territories (Petrides and Pandya, 2002; Gerbella et al., 2007). Following these injections, retrogradely labeled neurons formed discrete clusters not only in the ipsilateral and contralateral VLPFC but also in the ipsilateral DLPFC. Many of these clusters were observed across serial tangential sections spanning different cortical layers, suggesting they are radially oriented, columnar-like structures. The width of these clusters in both the VLPFC and DLPFC, as measured by their minor axis, was approximately 1.2 mm. Furthermore, our observation that afferent inputs to this VLPFC region from the superior temporal sulcus (STS) also originate from discrete clusters of projection neurons is consistent with an earlier report of patchy projections from the inferior temporal gyrus to VLPFC (Lima et al., 1990), hinting at a broader principle of clustered connectivity in temporo-prefrontal afferent pathways.

### 4.1 Clustered architecture of VLPFC connections: comparative organization and functional implications

While clustered or “patchy” connectivity is a well-established organizational principle in the cerebral cortex (Amir et al., 1993; Watakabe et al., 2023), our study provides novel insights by characterizing the specific architectural patterns of connections in the caudal VLPFC and contrasting them with those in other functionally distinct regions. Our primary finding is that a consistent, fine-grained clustered architecture is a fundamental feature of both the long-range ipsilateral and the interhemispheric callosal connections of the VLPFC, suggesting a common design principle for how information is handled in this region.

A key distinction of the VLPFC’s clustered architecture emerges when comparing it to that of the adjacent dorsolateral prefrontal cortex (DLPFC). Quantitatively, the discrete clusters we observed in the VLPFC exhibit a relatively symmetrical, ovoid morphology, with an average major-to-minor axis ratio of approximately 1.5:1. This morphology stands in marked contrast to the highly anisotropic, “stripe-like” organization of intrinsic connections documented in the DLPFC (Levitt et al., 1993; Kritzer and Goldman-Rakic, 1995; Pucak et al., 1996; Melchitzky et al., 1998). Those studies describe the DLPFC stripes as spanning several millimeters in length while being only 300–600 μm wide, resulting in a much larger aspect ratio (e.g., approximately 6.6:1) (Pucak et al., 1996). We propose that this difference in circuit architecture may be the anatomical substrate for the well-established functional dissociation between these two prefrontal regions. The VLPFC is primarily implicated in processing non-spatial information, such as object features and abstract rules (Wilson et al., 1993; Petrides, 2005), for which an architecture of discrete clusters may be advantageous for segregating and integrating distinct informational components. In contrast, the DLPFC is crucial for spatial working memory and the online manipulation of information (Goldman-Rakic, 1995; Tanji and Hoshi, 2008), functions that may be better served by a stripe-like architecture that could support computations across a continuous representation of space (Goldman-Rakic, 1995; Kritzer and Goldman-Rakic, 1995).

Our results also suggest a potential anteroposterior (AP) topographical organization within the connectivity of the caudal VLPFC. The observation that all clusters identified within the DLPFC originated exclusively from the most anterior one or two of our VLPFC injection sites could imply that these injections were situated within Area 12, which may have preferential connections to the DLPFC. Conversely, the lack of distinct distal clusters from the most posterior injection site might reflect the connectional properties of its putative location, Area 8r. Our quantitative analysis lends support to the idea of an AP gradient, showing that the AP position of an injection site significantly influenced both the density and the spacing of the resulting clusters. It must be emphasized, however, that these interpretations are speculative. The number of successful injections at each AP position was small, and our areal assignments were not based on cytoarchitectonics. Nonetheless, our findings provide a clear hypothesis for future studies to investigate the precise relationship between cytoarchitectonic subregions and the clustered connectional architecture in the VLPFC.

A key question in cortical organization is the extent to which connections are organized in relation to the vertical, columnar axis. Our depth profile analysis of the connectional clusters provides direct evidence on this matter for the VLPFC. We found that the majority of clusters (69%) were trans-laminar, spanning both the supragranular and infragranular layers. Furthermore, the quantitative properties (size and density) of these trans-laminar clusters remained consistent across their vertical extent (Figure 11), strongly suggesting that the clustered architecture we describe is not a phenomenon restricted to specific layers. Rather, these clusters represent radially oriented, columnar-like structures that engage neurons across different laminae, providing an anatomical substrate for integrating processing between superficial and deep cortical circuits. This trans-laminar nature, however, varied by region, being less prevalent in the ipsilateral DLPFC (50% of clusters) than in the VLPFC (85% of clusters), suggesting a difference in the mode of connectivity between these areas.

In contrast to these intrinsic and callosal connections, our finding that afferent projections from the STS originate predominantly from the supragranular layers is strongly consistent with a feedforward pathway from a temporal association area to the PFC (Felleman and Essen, 1991). This observation aligns well with extensive previous work on prefrontal-temporal connectivity. For instance, pathways from the superior temporal gyrus to various prefrontal areas have been shown to arise primarily from the supragranular layers, while the reciprocal, feedback projections from the prefrontal cortex terminate heavily in the superficial layers of the temporal cortex, particularly layer I (Barbas et al., 2005). These patterns conform to a general principle, often referred to as the “structural model,” where the laminar origin and termination of corticocortical connections can be predicted by the relative architectonic differentiation of the connected areas (Barbas, 1986; Barbas and Rempel-Clower, 1997; Rempel-Clower and Barbas, 2000). According to this model, pathways directed from less-differentiated cortices to more highly differentiated cortices typically originate in the deep layers (a feedback-like pattern), whereas pathways in the opposite direction, such as the temporo-prefrontal projections observed here, originate in the superficial layers (a feedforward-like pattern) (Barbas, 1986). Our findings therefore provide further support for these organizational rules governing connections between sensory association and higher-order prefrontal cortex.

Furthermore, our demonstration that contralateral callosal projections to the VLPFC are not only topographically organized (Schwartz and Goldman-Rakic, 1984; Yan et al., 2022) but also distinctly clustered offers another layer of functional insight. This arrangement contrasts with the callosal connections of primary sensory areas, such as the primary visual cortex (V1), where topographic projections are reportedly more diffuse rather than forming discrete clusters (Bosking et al., 2000). We suggest that the clustered nature of interhemispheric communication in the VLPFC represents a specialization of higher-order association cortex. This architecture would enable a precise and selective exchange of highly processed, non-spatial information between the hemispheres, a computational demand fundamentally different from that required in early sensory areas.

Another interesting finding of our study is that the properties of connectional clusters, particularly their neuronal density, are independent of the physical distance from their input source within the VLPFC (Figure 9). This lack of distance-dependence stands in interesting contrast to the organizational principles of horizontal connections in earlier stages of the visual hierarchy. For instance, our previous studies using anterograde tracers demonstrated that in the primary visual cortex (V1), the strength of terminal patch connections rapidly decreases with projection distance (Tanigawa et al., 2005; Wang et al., 2017). This distance-dependence was weaker in the higher-order visual area TE (inferior temporal cortex), where some strongly connected patches were found at distal locations. Our finding in the VLPFC, a higher-order association area, of a complete lack of correlation between cluster density and distance may represent a further step in this hierarchical trend. This suggests that as one moves up the cortical hierarchy from primary sensory to high-order association areas, the principles governing the strength of local connections may become progressively less constrained by physical distance and more influenced by other factors, such as the functional relationship between connected neuronal populations.

This clustered architecture likely provides the structural basis for the diverse integrative functions of the VLPFC. For instance, area 45A, a primary focus of our study, is known to integrate diverse inputs, including auditory and multisensory information related to communication signals (Gerbella et al., 2007, 2010; Romanski, 2007, 2012; Saleem et al., 2014). The clustered, laterally projecting connections we observed are well-suited to perform this function. While tangentially organized connections in V1 preferentially link columns with similar functional properties (Gilbert and Wiesel, 1989; Malach et al., 1993; Bosking et al., 1997), those in higher-order association areas often display a different organizational logic adapted to more complex integrative demands. Connections in area MT and the inferior temporal cortex (ITC), for example, have been shown to link columns with different feature selectivities, likely contributing to the construction of more complex stimulus representations (Malach et al., 1997; Hu et al., 2025). It is therefore plausible that the VLPFC clusters similarly serve to bind and integrate diverse inputs—such as the visual and auditory components of a vocalization—into coherent representations. On a larger scale, these ∼1.2 mm anatomical clusters may serve as fundamental building blocks for the millimeter-scale functional domains, such as the category-selective patches identified with fMRI (Tsao et al., 2008; Haile et al., 2019), thereby providing a multi-level anatomical substrate for the complex cognitive functions of the VLPFC.

Our findings on the clustered organization of VLPFC connectivity also complement recent large-scale, single-neuron projectome studies in the macaque PFC. For instance, a recent study reconstructing the whole-brain axonal projections of individual PFC neurons revealed that their long-range projections, while divergent, terminate in highly specific and confined terminal fields (Gou et al., 2025). This established a principle of specificity at the output stage of PFC connections. Our results provide a critical, complementary perspective from the input side. We demonstrate that the population of neurons providing input to a focal VLPFC site is not diffusely scattered, but is itself organized into discrete, spatially segregated clusters. This finding reveals specificity at the input stage of PFC connectivity. Moreover, high specificity has also been demonstrated for contralateral projections targeting homologous cortical areas (Gou et al., 2025), a finding that aligns well with the topographically organized, clustered callosal projection system we observed. Taken together, these two methodologically distinct approaches converge on a common principle: PFC connectivity, viewed from either the origin or the termination, is not random but is highly specific. The combination of specific input sources (our clusters) and specific output targets, such as the terminal fields described in the projectome study by Gou et al. (2025) strongly implies the existence of dedicated pathways linking discrete neuronal populations. The presence of numerous such clusters, as shown in our work, therefore suggests that the broader association network is composed of many of these specific pathways operating in parallel, forming distinct processing channels.

### 4.2 Limitations, future directions, and conclusion

Several limitations should be acknowledged, alongside future directions. Firstly, the use of tangential sections, while advantageous for visualizing widespread connections, made precise correlation with cytoarchitectonic boundaries challenging (Gerbella et al., 2007), meaning injections targeting area 45A might have involved adjacent regions (Areas 8r, 12). It should also be noted that while we describe long-range ipsilateral clustered connectivity, the precise axonal trajectories of these connections were not traced in this study. Therefore, we cannot definitively exclude the possibility that some of the most distant connections might involve association fibers traversing the underlying white matter, rather than being strictly intracortical horizontal projections in the traditional sense. Secondly, the observed VLPFC clusters contrast with DLPFC stripes; while tracer type (retrograde vs. anterograde) might contribute (Levitt et al., 1993; Melchitzky et al., 1998), retrograde studies in DLPFC also revealed a highly organized, non-uniform distribution of labeled cells (Kritzer and Goldman-Rakic, 1995; Pucak et al., 1996), suggesting tracer type alone is not a full explanation. A direct comparative study in both regions with identical methods would be valuable. Future research combining high-resolution tracing with functional methods (electrophysiology, fine-scale fMRI) is essential to link these anatomical VLPFC clusters to specific functions (e.g., responses to faces, communication signals) (Sugihara et al., 2006; Tsao et al., 2008; Haile et al., 2019). Examining the developmental emergence of this clustered organization and its relation to broader VLPFC connectivity networks (Borra et al., 2011; Yeterian et al., 2012; Kravitz et al., 2013; Saleem et al., 2014) will also be crucial. In conclusion, despite these limitations, this study provides clear anatomical evidence for a fine-scale, clustered architecture governing its laterally projecting ipsilateral and interhemispheric connections within the macaque caudal VLPFC, offering a new perspective on the structural basis for information processing in this critical associative cortical region.

## Data Availability

The raw data supporting the conclusions of this article will be made available by the authors, without undue reservation.
