# Supplementary material for: Clustered architecture of ipsilateral and interhemispheric connections in macaque ventrolateral prefrontal cortex
Source: Front Neural Circuits. 2025 Aug 26;19:1635105. doi: 10.3389/fncir.2025.1635105 (PMC12417498; doi:10.3389/fncir.2025.1635105)
Supplement: Supplementary file 1 [file Data_Sheet_1.pdf]

Supplementary Material

Supplementary Figures

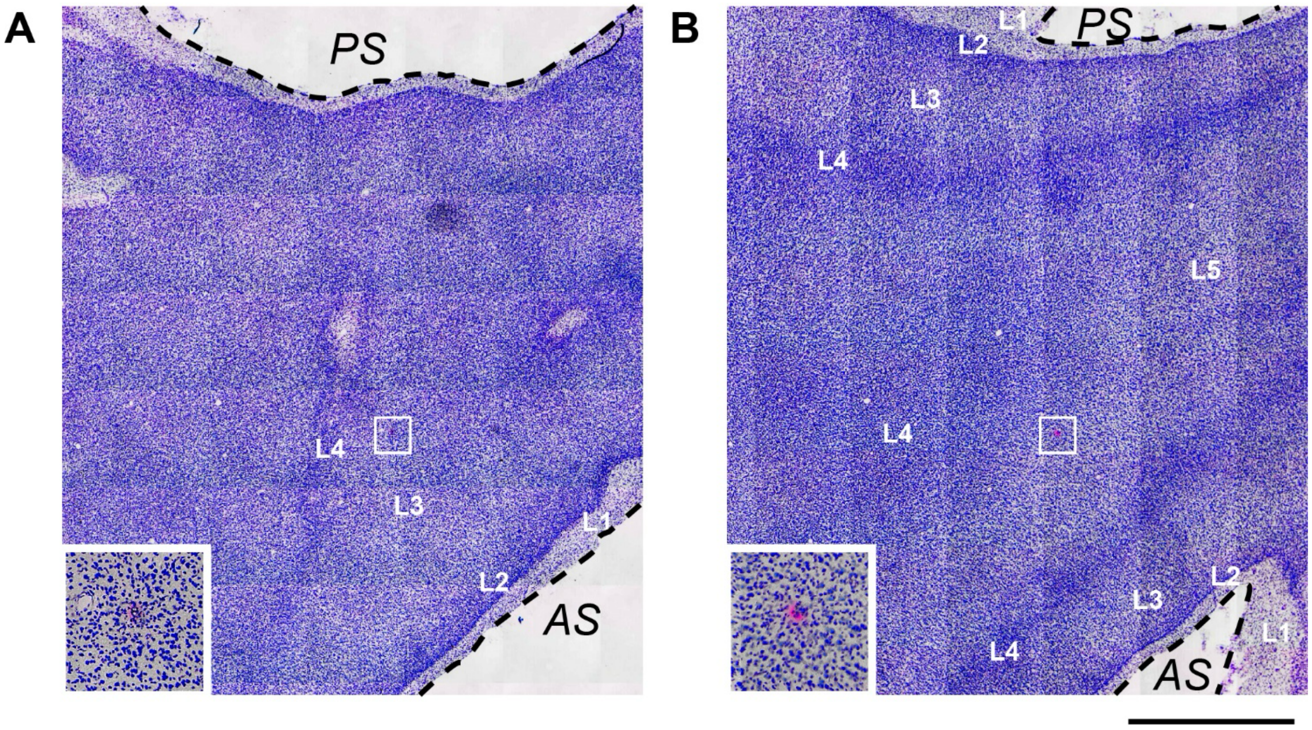

Supplementary Figure 1.

Representative Nissl-stained tangential sections from the left VLPFC of Case 1, illustrating cytoarchitectonic features used for laminar identification. (A) Section at an approximate depth of 200 µm from the pial surface, primarily showing supragranular layers (Layers II/III). The inset (bottom left) shows a higher magnification of the boxed region, which includes a CTB-555 injection site, revealing the tracer deposit. AS, arcuate sulcus; PS, principal sulcus. (B) Section at an approximate depth of 920 µm from the pial surface, primarily showing infragranular layers (Layers V/VI). The inset (bottom left) shows a higher magnification of the boxed region, which includes the same injection site shown in (A). Cortical layers are indicated by Roman numerals. Scale bars: 2 mm.

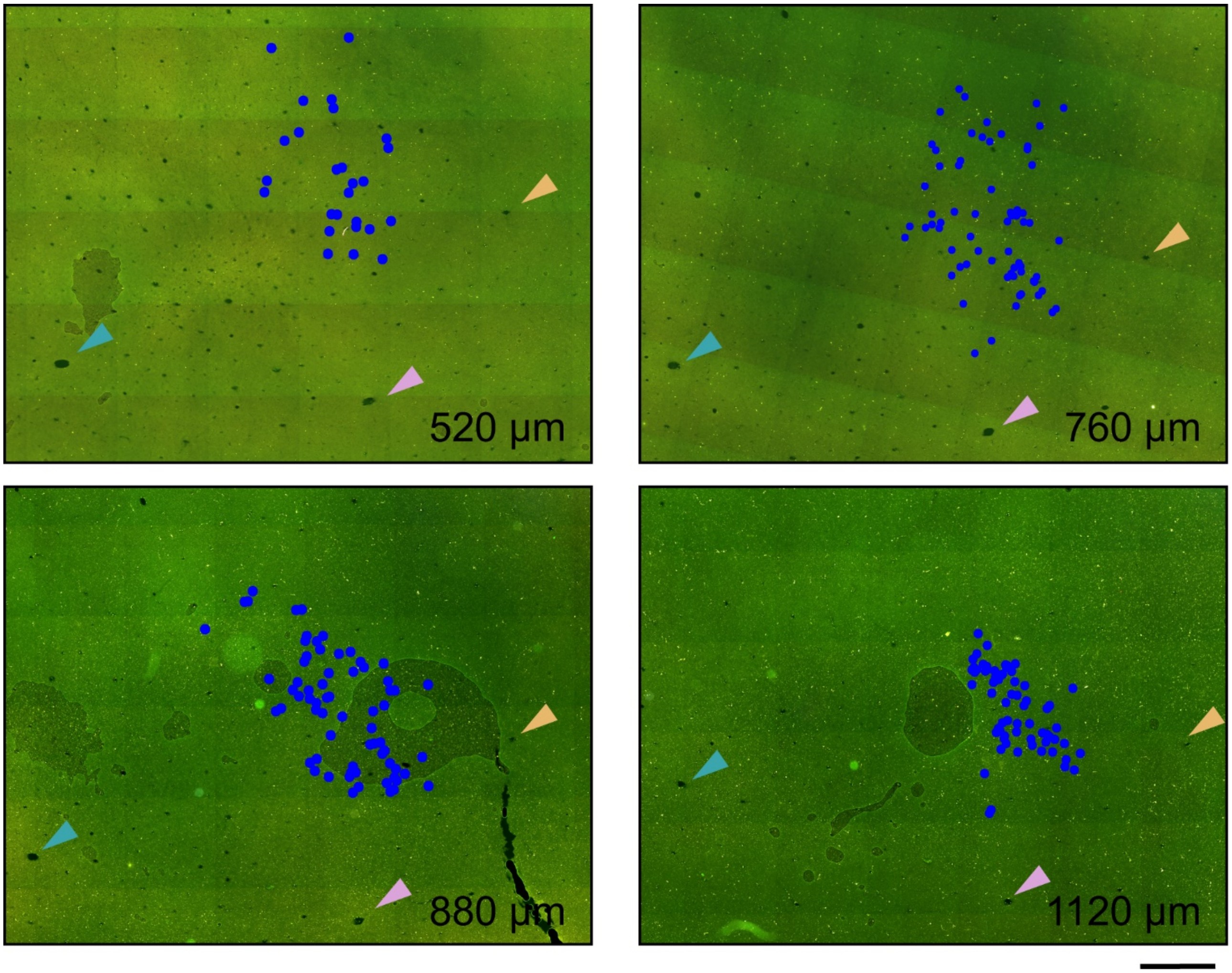

Supplementary Figure 2.

Photomicrographs illustrating the identification of a single neuronal cluster across four serial tangential sections using vascular landmarks. The four panels show the same tangential location at four different depths from the pial surface (indicated in µm). Blue dots represent retrogradely labeled neurons (CTB-647). Colored arrowheads point to the consistent patterns of radial blood vessels that serve as fiducial markers, confirming that the neuronal aggregate in each panel belongs to the same cluster. Scale bar: 200 µm.

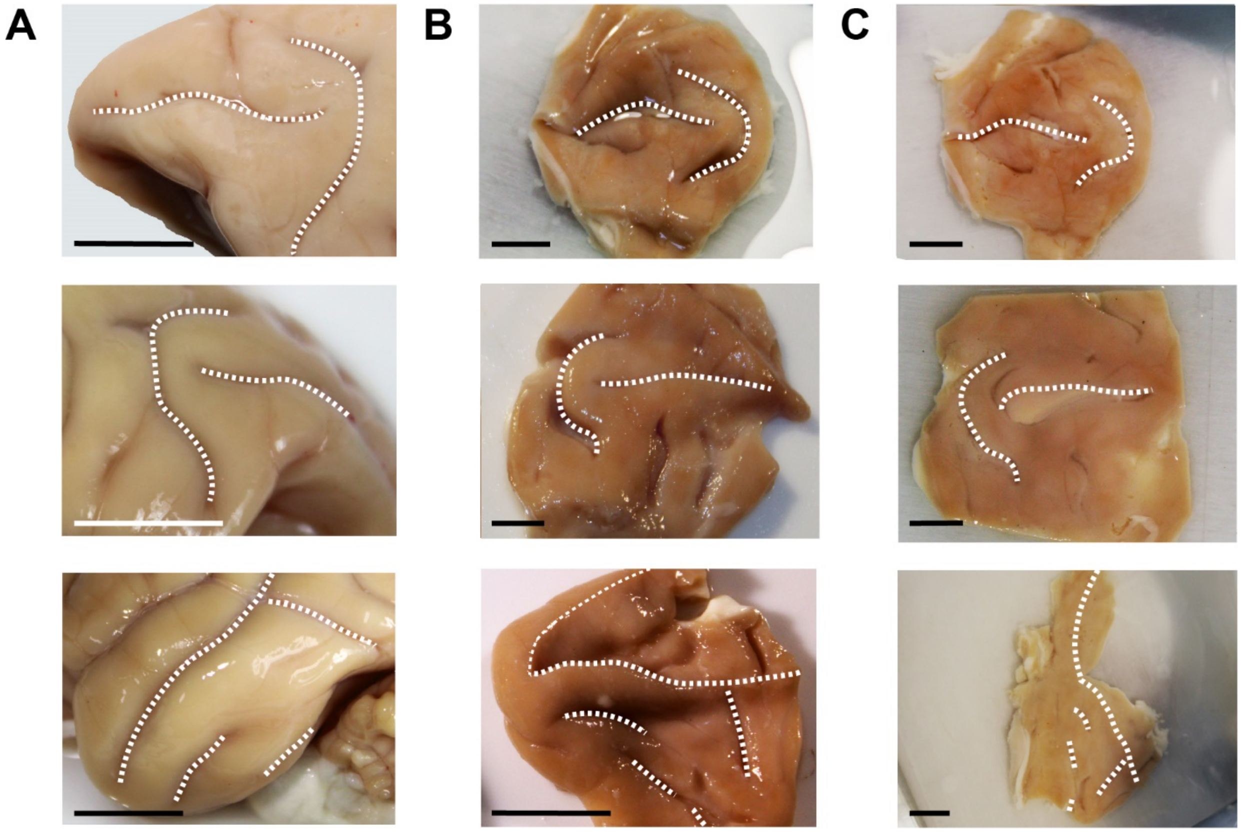

Supplementary Figure 3.

Photographs illustrating the stages of brain dissection and cortical flattening for Case 1. (A) Portions of the brain prior to being blocked, after perfusion and extraction, including (from top to bottom) the left prefrontal cortex (PFC), right PFC, and left temporal lobe. (B) The corresponding brain blocks undergoing unfolding to expose sulcal cortex. (C) The corresponding cortical tissue after being physically flattened between glass slides. Dashed lines delineate major sulci. Scale bars: 1 cm.

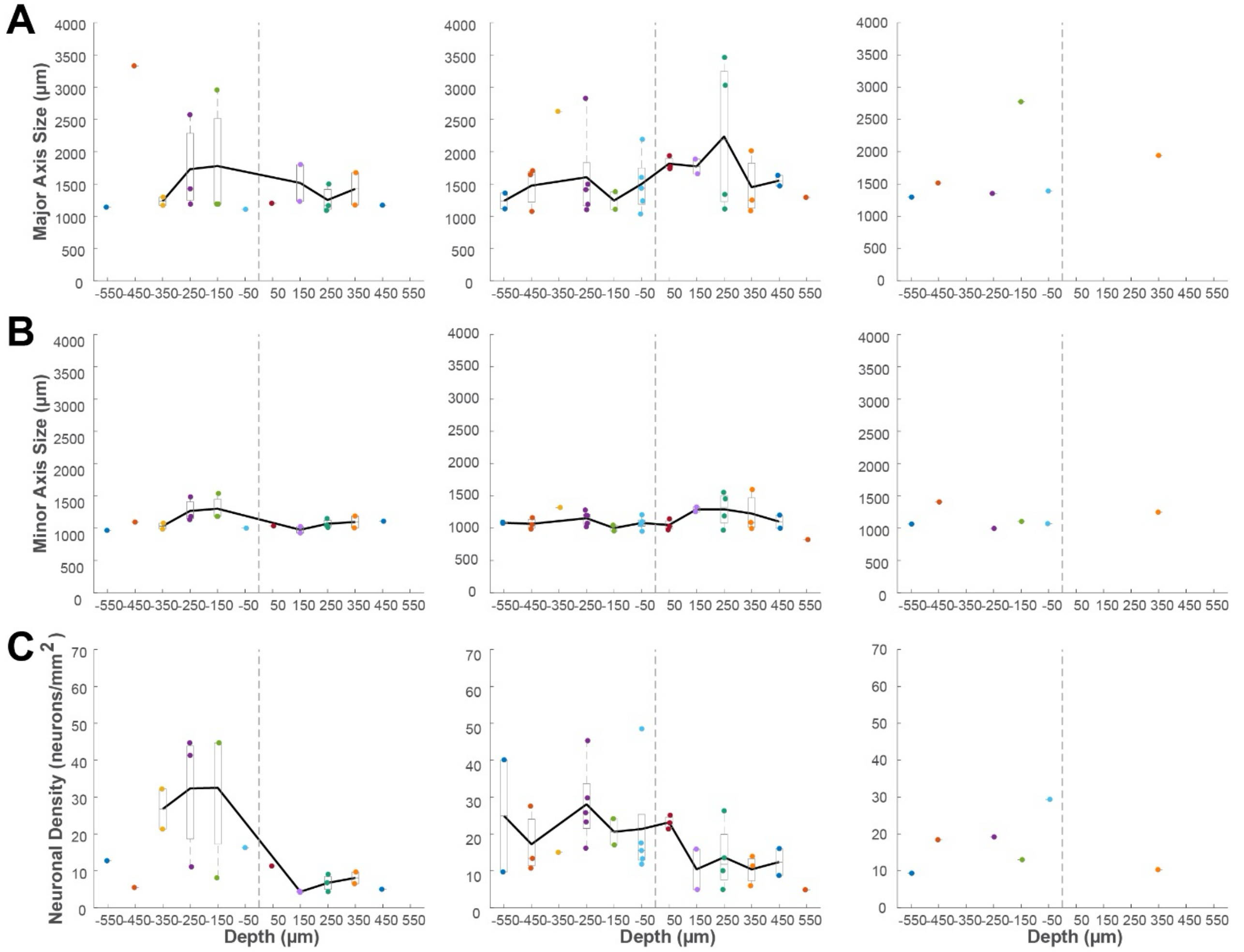

Supplementary Figure 4.

Histograms illustrating the quantitative properties of clusters identified in the ipsilateral DLPFC (left), contralateral VLPFC (middle), and contralateral DLPFC (right) as a function of cortical depth relative to layer 4. The solid black line in each plot connects the mean values for depth bins containing two or more data points. (A) Average major axis size (µm). (B) Average minor axis size (µm). (C) Average neuronal density (neurons/mm²). Conventions for the box plots are as described for Figure 8.
